# Modular Design for Proteins Assembling into Antifouling Coatings: Case of Gold Surfaces

**DOI:** 10.1021/acs.langmuir.3c00389

**Published:** 2023-06-27

**Authors:** Chuanbao Zheng, Nicolò Alvisi, Robbert Jan de Haas, Zhisen Zhang, Han Zuilhof, Renko de Vries

**Affiliations:** † Physical Chemistry and Soft Matter, Wageningen University & Research, Stippeneng 4, Wageningen 6708 WE, The Netherlands; ‡ Laboratory of Organic Chemistry, Wageningen University & Research, Stippeneng 4, Wageningen 6708 WE, The Netherlands; § Research Institute for Biomimetics and Soft Matter, Fujian Provincial Key Laboratory for Soft Functional Materials Research, Department of Physics, Xiamen University, Xiamen 361005, China; ∥ School of Pharmaceutical Sciences and Technology, Tianjin University, 92 Weijin Road, Tianjin 300072, China

## Abstract

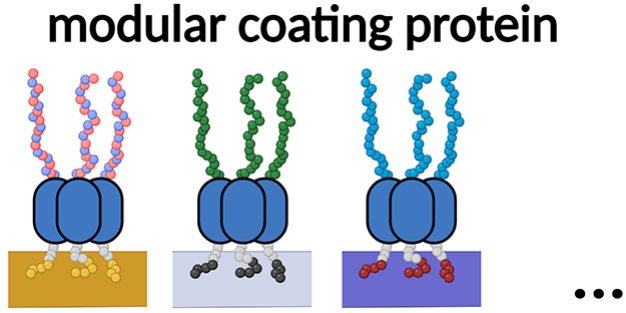

We analyze modularity for a ***B-M-E*** triblock protein designed to self-assemble into antifouling coatings. Previously, we have shown that the design performs well on silica surfaces when ***B*** is taken to be a silica-binding peptide, ***M*** is a thermostable trimer domain, and ***E*** is the uncharged elastin-like polypeptide (ELP), ***E*** = (GSGVP)_40_. Here, we demonstrate that we can modulate the nature of the substrate on which the coatings form by choosing different solid-binding peptides as binding domain ***B*** and that we can modulate antifouling properties by choosing a different hydrophilic block ***E***. Specifically, to arrive at antifouling coatings for gold surfaces, as binding block ***B*** we use the gold-binding peptide GBP1 (with the sequence MHGKTQATSGTIQS), while we replace the antifouling blocks ***E*** by zwitterionic ELPs of different lengths, ***E^*Z*^***_*n*_ = (GDGVP-GKGVP)_*n*/2_, with *n* = 20, 40, or 80. We find that even the ***B-M-E*** proteins with the shortest ***E*** blocks make coatings on gold surfaces with excellent antifouling against 1% human serum (HS) and reasonable antifouling against 10% HS. This suggests that the ***B***-***M***-***E*** triblock protein can be easily adapted to form antifouling coatings on any substrate for which solid-binding peptide sequences are available.

## Introduction

Interfacing synthetic materials with living organisms, cells, or biological fluids is required in many technologies, from in vitro biosensing to in vivo biomaterial implants, and often requires combining different disciplines such as materials science and bioengineering.^1−4^ In many cases, it is crucial to obtain control over the interactions between the surface of a solid material and a liquid containing biological molecules. A key approach for controlling the interactions of surfaces with biomolecules is the application of a surface coating that can modulate the interactions with the biomolecules and in this way remedy the shortcomings of using the bare surface, such as undesired adsorption from biomacromolecules or microorganisms.

In many cases, interfacing synthetic materials with living organisms boils down to first preventing any unwanted interactions of the synthetic surface with the adjacent biological fluid (“antifouling”) and then adding the desired specific interactions required by the application. Therefore, the antifouling coating preventing non-specific interactions of the surface with biomolecules, microorganisms and cells, have been investigated in many studies.^5−12^ Both chemical and physical attachments of the coatings have been explored, with chemical attachment—either grafted from brushes or grafted to brushes^13^—generally leading to more stable coatings of higher functionality,^14^ while physical attachment generally allows for simpler and more easily scalable coating processes. Particularly, successful antifouling coatings consist of brushes of uncharged hydrophilic or zwitterionic flexible polymers, and detailed systematic studies thereof exist.^15^ For example, well-known uncharged antifouling polymers are polyethylene glycol (PEG)^16−19^ and poly (*N*-(2-hydroxypropyl) methacrylamide).^20^ Brushes of the PEG polymer can be chemically grafted onto surfaces with high density and in this way promote interface hydration which prevents biomolecular adsorption.^21,22^ Also, zwitterionic polymers, such as polycarboxybetaine methacrylate, have been shown to lead to excellent antifouling when grafted as a brush.^23^

Using synthetic materials allows for tailor-made functionalization, but the synthesis of the required components typically has a larger ecological footprint than required for the in vivo synthesis of biomacromolecules with similar functions. Biomolecules, in particular proteins, can be precisely manipulated at the DNA level. Very large peptide and protein libraries can be generated for screening potential interactions with natural materials. In fact, surface-binding peptides selected from various types of libraries have proven to be useful tools in materials science,^24^ with peptides having been isolated that bind strongly to different surfaces, such as metals,^25,26^ minerals,^27,28^ plastics,^29,30^ and even semiconductors.^31^

Previously,^32^ we have argued that protein design should, in principle, allow for the creation of proteins that self-assemble into antifouling polypeptide brushes that are not only easy to apply but also highly stable and functional. This earlier work considered silica as a model surface. A ***B***-***M***-***E*** modular design consisting of three domains was proposed, where ***B*** is a solid-binding peptide for anchoring to the surface, ***M*** is a multimerization domain for increasing the overall protein binding strength to the surface through multivalency, and ***E*** is an uncharged hydrophilic elastin-like polypeptide (ELP)^33,34^ serving as an antifouling block. Compared to first-generation coatings with the simpler ***B***-***E*** design,^32,35^ the multivalent ***B***-***M***-***E*** designs assembled into highly stable coatings that could not be displaced, neither by high salt buffers nor by serum albumin protein solutions. Also, the coatings were highly antifouling against high concentrations of serum albumin. While we hypothesized that the ***B***-***M***-***E*** protein design should be highly modular and allow for swapping out binding domains ***B*** and antifouling domains ***E*** to lead to coatings with different functionalities for different materials, we did not demonstrate this in our previous study.

Therefore, in the current work, we explicitly explore the modularity of the ***B***-***M***-***E*** design by testing it with binding blocks ***B*** for gold as opposed to silica and for antifouling polypeptides ***E*** of different lengths and with different sequences. Gold is chosen as a model surface since it is chemically very different from silica, is an important surface material in biosensing, and a number of well-characterized solid-binding peptides are available for gold surfaces. To test the antifouling properties, we here more stringently challenge the coatings with human serum (HS) of various dilutions, as opposed to only single-protein serum albumin solutions.

To replace the original silica-binding domain, we chose a gold-binding peptide that has been extensively studied both by experimental work and by computer simulations,^36−38^ and which has previously been referred to as GBP1,^26^ a non-cysteine peptide with a single-letter amino acid sequence MHGKTQATSGTIQS. Previously,^32^ we have used ***E*** = (GSGVP)_40_ for the antifouling domain. Here, we explore a series of three different lengths of the zwitterionic ELP sequence, ***E*** = ***E***^***Z***^_*n*_ = (GKGVP-GDGVP)_*n*/2_ with *n* = 20, 40, or 80.

## Materials and Methods

### Construction of Expression Plasmids for Polypeptides

All protein constructs used in this study carry a His-tag (six repeats of histidine, H_6_) for later nickel affinity purification. The three ***B***-***M***-***E*** protein constructs in this study are abbreviated as ***B***-***M***-***E***_*n*_, where *n* = 20, 40, or 80 is the number of pentapeptide repeats of the ***E*** block.

Previously,^32^ we have produced the silica-binding ***B***-***M***-***E*** protein design H_6_-***B***^RT^-***E***^*S*^_3_-***M***^HR00C_3_2^-***E***^*S*^_40_, where ***B***^RT^ was a sequence of a silica-binding peptide, and the ***E*** block consisted of repeats of the elastin-like pentapeptides with serine (S) as the guest residue, ***E*** = ***E****^S^*_40_ = (GSGVP)_40_. The multimerization domain ***M***^HR00C_3_2^ was a thermostable trimer previously *de novo* designed and characterized by Fallas et al.^39^ A short ***E***^*S*^_3_ linker, ***E***^*S*^_3_ = (GSGVP)_3_, was used to connect the solid-binding peptide to the trimerization domain. Plasmids used in the construction of previous silica proteins are used here to construct the gold-binding versions, H_6_-***B***^GBP1^-***E***^*S*^_3_-***M***^HR00C_3_2^-***E***^*Z*^_20_, H_6_-***B***^GBP1^-***E****^S^*_3_-***M***^HR00C_3_2^-***E***^*Z*^_40_ and H_6_-***B***^GBP1^-***E***^*S*^_3_-***M***^HR00C_3_2^-***E***^*Z*^_80_, where ***B***^GBP1^ = GBP1 = MHGKTQATSGTIQS is the gold-binding peptide sequence. All the plasmids mentioned above contain necessary features for recursive directional ligation by plasmid reconstruction (PRe-RDL) cloning, as described by McDaniel et al.^40^

A gene fragment encoding GBP1, with suitable overhangs for Gibson assembly,^41^ was purchased from Integrated DNA Technologies (IDT, Leuven, Belgium). Gibson assembly was used to insert this gene fragment into the linearized plasmid for H_6_-***B***^RT^-***E***^*S*^_3_-***M***^HR00C_3_2^, yielding an expression plasmid for H_6_-***B***^GBP1^-***E***^*S*^_3_-***M***^HR00C_3_2^. Primers used for obtaining the linearized plasmid for H_6_-***B***^RT^-***E***^*S*^_3_-***M***^HR00C_3_2^ from a previously designed plasmid^32^ are given in Table S1. The DNA and amino acid sequences for the gene fragment encoding ***B***-***M***-***E*** proteins are shown in Tables S1 and S2*.*

PRe-RDL was used to obtain oligomers of the genes for ***E***^*Z*^_40_ and ***E***^*Z*^_80_. A plasmid encoding ***E***^*Z*^_**20**_ flanked by sequences necessary for gene oligomerization via PRe-RDL was purchased from TwistBioscience. Restriction enzymes and DNA modifying enzymes for the PRe-RDL procedure were purchased from New England Biolabs.

For duplication of the ***E***^*Z*^_20_ gene to obtain the ***E***^*Z*^_40_ gene using PRe-RDL, the plasmid for ***E***^*Z*^_20_ gene was digested with AcuI and BglI. The same plasmid was also digested with BseRI and BglI. The two digested fragments were ligated to obtain the ***E***^*Z*^_40_ plasmid. The gene for ***E***^*Z*^_40_ was duplicated in the same above-mentioned way using PRe-RDL to obtain the plasmid with the ***E***^*Z*^_80_ gene.

Next, the plasmid for H_6_-***B***^GBP1^-***E***^S^_3_-***M***^HR00C_3_2^ was digested using AcuI and EcoRV, and plasmids containing genes for ***E***^*Z*^_20_, ***E***^*Z*^_40_, and ***E***^*Z*^_80_ were digested with BseRI and EcoRV. Pairs of digested fragments were ligated to obtain plasmids encoding for H_6_-***B***^GBP1^-***E***^*S*^_3_-***M***^HR00C_3_2^-***E***^*Z*^_20_, H_6_-***B***^GBP1^-***E***^*S*^_3_-***M***^HR00C_3_2^-***E***^*Z*^_40_, and H_6_-***B***^GBP1^-***E***^*S*^_3_-***M***^HR00C_3_2^-***E***^*Z*^_80_.

### Protein Expression and Protein Purification

All plasmids were sequenced before starting protein expression. Plasmids containing the desired DNA sequences were transformed into T7-Express*Escherichia coli* (New England Biolabs, USA). The transformed strains were cultivated in 250 mL Erlenmeyer flasks with 25 mL of terrific broth medium containing 50 μg/mL kanamycin at 37 °C/215 rpm for at least 16 h as the start culture. The start culture was diluted in a 2 L Erlenmeyer flask with up to 1 L of autoclaved lysogeny broth (LB) medium (tryptone 10 g/L, NaCl 10 g/L, and yeast extract 5 g/L). When the culture OD_600_ reached 0.6–0.8, IPTG (isopropylthio-β-galactoside) was added to LB at a final concentration of 1 mM. Next, bacteria were incubated for protein expression at 18 °C/215 rpm > 21 h before harvesting. After overnight protein expression, cultures were centrifuged at 4 °C/6000 rpm for 30 min to pellet the cells. Bacterial pellets were resuspended in 30 mL of cold lysis buffer (50 mM Tris pH 8.0, 300 mM NaCl, and 30 mM imidazole). Then, 300 μL of PMSF (phenylmethylsulfonyl fluoride) was added to the bacteria containing lysis buffer to a final concentration of 1 mM. Next, resuspended cells were sonicated using a Q125 Sonicator (Qsonica) with a 2 s on/off duty cycle at 85% amplitude. After sonication, the bacterial lysate was centrifuged at 4 °C and 30 000 × *g* for 30 min to obtain a supernatant with soluble proteins. Overexpressed proteins were isolated from the supernatant using gravity-immobilized metal-ion affinity chromatography (IMAC) columns (Bio-Scale Mini Profinity IMAC cartridge, Bio-Rad Laboratories, USA, column volume = 5 mL). Before elution, the column was washed with 10 column volumes of lysis buffer. Elution was done using 1 column volumes of elution buffer (50 mM Tris pH 8.00, 300 mM NaCl, and 300 mM imidazole). A final polishing step was done using size exclusion chromatography (SEC). 1 mL of samples from IMAC purification was filtered using a 0.22 μm filter (Millex-GV, Sigma) and then injected into a Superdex 200 Increase 10/300GL column (GE Healthcare). The SEC purification process was performed with a flow rate of 0.75 mL/min in phosphate-buffered saline (PBS) buffer pH 7.4 on a 1260 Infinity II HPLC (Agilent). The purity of the proteins throughout the purification process was monitored using SDS-PAGE (sodium dodecyl sulfate polyacrylamide gel electrophoresis). Analytical SEC of purified proteins was performed on a Superose S6 10/300 gl (GE Healthcare) column with a flow rate of 0.5 mL/min.

### Matrix-Assisted Laser Desorption/Ionization Time of Flight Mass Spectrometry

The molecular masses of the proteins were verified using matrix-assisted laser desorption/ionization time of flight (MALDI-TOF) mass spectrometry. All samples were desalted before measurement using a dialysis device (Thermo Scientific Slide-A-Lyzer MINI) with a cut-off of 3.5 kDa. Then, the dialyzed protein samples were concentrated to 1mg/mL. Next, matrix solutions for measurement are prepared as follows: 5 mg of DHB (2,5-dihydroxybenzoic acid) was dissolved in 200 μL of solution (133.3/66.6, v/v, Milli-Q water/0.1% formic acid in acetonitrile). Then, 1 μL of the matrix was placed on a target plate (MTP 384 target plate ground steel T F, Bruker), followed by 1 μL of protein solution. After that, the mixed samples were gently dried using a hair dryer. Mass spectra were obtained using a Bruker UltraFlextreme (Bruker Daltonics). The data were processed using Bruker FlexAnalysis (version 3.4).

### Circular Dichroism Analysis

A Jasco Spectropolarimeter J-715 was used to record circular dichroism (CD) spectra. Samples were dialyzed to Milli-Q before measurement using a dialysis device (Thermo Scientific Slide-A-Lyzer MINI) with a cut off of 3.5 kDa. All samples prior to CD measurements were diluted to 0.1 mg/mL and placed into a sonication bath for 10 min to minimize potential aggregation. All measurements were performed in a quartz cuvette (QS 110-1-40, Hellma Analytics) with a 1 mm path. For spectra, the continuous scanning mode was used, with a wavelength step size of 0.1 nm, a scanning speed of 50 nm/min, and a bandwidth of 2 nm. Spectra shown are averages of 15 acquired spectra. For temperature ramp measurements, ellipticity at 222 nm was monitored continuously from 20 to 95 °C at a rate of 1 °C/min.

### Quartz Crystal Microbalance with Dissipation Monitoring

Gold-coated quartz sensors (QS-QSX301) were purchased from Biolin Scientific, Sweden. QCM data was obtained using a Q-Sense E4 QCM-D instrument (Biolin Scientific, Sweden). ***B-M-E*** protein solutions were diluted to 10 μM in PBS buffer, then filtered with a 0.22 μm pore size filter, and placed in a sonication bath for 10 min prior to use. For measurement, both protein coating formation and antifouling test were performed at a flow rate of 50 μL/min. First, a stable quartz crystal microbalance with a dissipation monitoring (QCM-D) baseline was obtained by prolonged flushing of the QCM-D channels. This was done until frequency variations were less than ∼2 Hz. Next, the gold-binding ***B***-***M***-***E*** protein was flushed onto the sensors for 30 min, followed by a 15 min PBS wash step. Finally, for analyzing the antifouling behavior of the coating, bovine serum albumin (BSA, 1 mg/mL in PBS), 1% HS, and 10% HS were injected for 30 min, followed by 15 min of PBS wash step. The QCM-D data was analyzed using the QSense Dfind version 1.2.7. (Biolin Scientific, Sweden).

### Atomic Force Microscopy and X-ray Photoelectron Spectroscopy

Commercial gold sensor chips (dimensions: 12 × 7 mm) were used as an atomic force microscopy (AFM) substrate. Gold sensor chips are traditional glass slides with a 45–50 nm gold thin film deposited onto an adhesion layer (Plasmetrix Technologies Inc, Canada). Gold surfaces were first plasma-cleaned followed by extensive rinsing using Milli-Q water and drying using nitrogen gas. To prepare samples for AFM imaging, 50 μL of the 0.5 μM and 5 μM ***B****-****M***-***E***_20_ protein in PBS was applied to the clean gold surface and then left to incubate for 3 s and 5 min, respectively. After incubation, the samples were again rinsed with MQ water and dried using nitrogen gas. Next, the gold sensors were imaged using both a Multimode AFM (Bruker, California) with the ScanAsyst imaging mode in air and X-ray photoelectron spectroscopy (XPS). Scanasyst Air cantilevers (Bruker) were used with the following specifications: thickness 650 nm, length 115 μm, width 25 μm, resonance frequency 70 kHz, and spring constant 0.4 N/m. Data was analyzed by NanoScope Analysis version 1.5 (Bruker). XPS measurements were performed using a JPS-9200 photoelectron spectrometer (JEOL Ltd., Japan) with a focused monochromated Al Kα X-ray source (spot size of 300 μm) radiation at 12 kV and 20 mA, with 10 eV as the analyzer pass energy.

### Dynamic Light Scattering

A ZS-Nano (Malvern, UK) instrument with a scattering angle of 173° was used to measure the hydrodynamic size and zeta potential of gold nanoparticles (GNPs) before and after the protein coating. GNPs were purchased from Sigma-Aldrich (60 nm, OD 1, stabilized suspension in citrate acid). According to the product information provided by the manufacturer (CytoDiagnostics, Inc.), the concentration of gold nanoparticles is 1.9 × 10^9^ GNPs/mL. Protein samples in PBS buffer at 0.1 μM were filtered using a 0.22 μm pore size filter. The protein samples were placed into a sonication bath for 10 min prior to use. The protein samples and GNPs were mixed in a series of protein volumes: GNPs with a volume ratio of 1:49, 5:45, 15:35, 25:25, or 35:15, each time reaching a final volume of 50 μL. Protein:GNP mixtures were incubated for 5 min before starting the measurements. All measurements were performed in a quartz cuvette (105.251.005-QS, Hellma Analytics) with a light path of 3 mm at 20 °C. Each reported particle size is an average value of 15 independent measurements. The reported hydrodynamic sizes were obtained using the Zetasizer software version 7.13 (Malvern, U.K.). The reported values were obtained from a distribution fit performed by the Zetasizer analysis software. In all cases, a single peak dominated the scattering intensity, and values for this peak are reported.

## Results and Discussion

### Protein Design, Production, and Characterization

**Figure 1:**
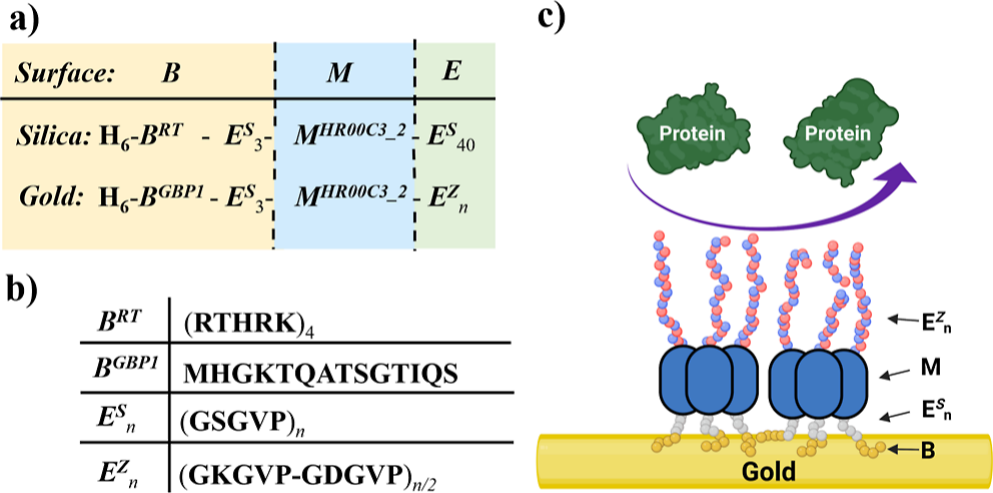
Design of gold coating ***B***-***M***-***E*** proteins. (a) ***B-M-E*** construct for our previous silica-binding design^32^ and current gold-binding design. (b) Single-letter amino acid of different parts of ***B-M-E*** constructs. ***B***^***RT***^ and ***E***^***S***^_***n***_ are the silica-binding peptide and ELP sequences used in a previous study;^32^***B***^***GBP1***^ and ***E***^*Z*^_*n*_ are the gold-binding peptide and ELP sequences used in this paper. (c) Schematic representation of the ***B-M-E***_***20***_ triblock, adsorbed on the gold surface and antifouling against approaching proteins. ***B*** = ***B***^***GBP1***^, where ***M*** = ***M***^***HR00C3_2***^ is from Fallas et al.^39^ and ***E*** = ***E***^*Z*^_20_.

In our previous study,^32^ we designed a ***B***-***M****-****E*** triblock protein that spontaneously assembled on silica surfaces into coatings with good antifouling properties. Here, we redesigned the silica coating protein to instead coat gold surfaces. A precise domain organization of the sequences of the original silica-coating protein and of the sequences for the redesigned gold coating proteins are shown in Figure 1a, with a legend for the sequences of the different blocks (Figure 1b), and a schematic representation of the antifouling action of a gold-bound ***B***-***M***-***E*** protein shown in Figure 1c.

We designed a series of gold-coating proteins with increasing lengths of the antifouling block that we here refer to as ***B***-***M***-***E***_20_, ***B***-***M***-***E***_40_, and ***B***-***M***-***E***_80_ (unless specified, in this work, ***B***-***M***-***E*** refers to the protein with ***E*** = ***E***^***Z***^), where the length is expressed in terms of the number of elastin-like pentapeptide repeats to arrive at the new designs. We replaced the silica-binding sequence ***B***^*RT*^ in the original design by the gold-binding sequence ***B***^*GBP1*^ and replaced the original elastin-like antifouling block with serine (S) as a host residue in the elastin-like pentapeptide motif, ***E***^*S*^_40_, with a zwitterionic elastin-like block ***E^Z^***_*n*_ with alternating aspartic acid (D) and lysine (K) as guest residues and the number of pentapeptide repeats *n* = 20, 40, or 80.

In our new designs, the surface anchor ***B***^*GBP1*^ is a 14-amino-acid gold-binding peptide referred to as GBP1.^26^ Many gold-binding peptides have been reported in the literature,^26,42,43^ but GBP1 is particularly well characterized both experimentally and with computer simulations.^37,38,44−47^ Also, GBP1 could bind to gold surfaces even at high salt concentrations.^48^ Moreover, GBP1 is a peptide without cysteine (C) residues. In previous studies,^49−51^ peptides or polymers containing a sulfhydryl group (-SH) were used to form a thiol bond with gold atoms to anchor peptides or proteins on gold surfaces. Here, we chose a cysteine-free peptide as a surface anchor to show the potential of the ***B-M-E*** construct as a non-covalent coating. Unless specified, the ***B*** mentioned in below text is ***B***^*GBP1*^.

As in our previous design,^32^ the multimerization domain ***M*** is a well-characterized thermostable trimer previously computationally designed by Fallas et al.^39^ and referred to as *HR00C_3_2* (PBD ID: 5K7V).

Recent studies have linked the strong interaction between zwitterionic polymers and water molecules with their good antifouling properties.^52−55^ Zwitterionic peptides and polypeptides have also been demonstrated to have good antifouling properties. For example, repeats(single-letter amino acid) of EK, DK, ER, and DR have been used in antifouling peptides for gold surfaces.^50^ Also, zwitterionic ELPs with repeated sequences of VPKEG have been shown to have very low interactions with blood proteins.^56^ For the antifouling domain, an improvement was sought by using a zwitterionic ELP sequence with guest residues of the elastin-like pentapeptide being alternately D and K, rather than an uncharged hydrophilic sequence with the guest residue S. Here, we explore three zwitterionic polypeptide sequences of type ***E***^*Z*^_*n*_ = (GDGVP-GKGVP)_*n*/2_, for *n* = 20, 40, or 80.

**Figure 2:**
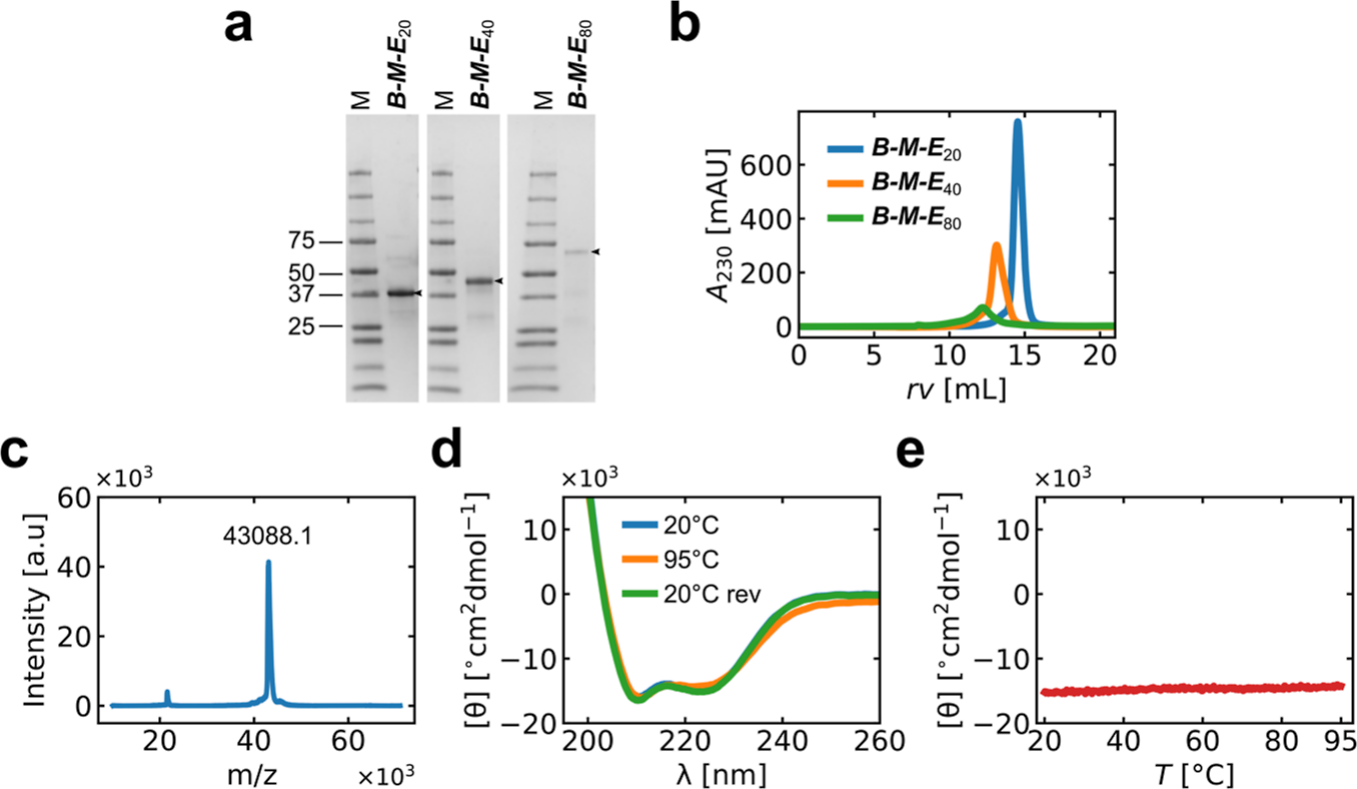
Protein purification and characterization. (a) SDS-PAGE analysis of purified ***B-M-E*** proteins. Arrows indicate bands corresponding to the purified proteins. (b) Analytical SEC of purified ***B-M-E*** proteins. Absorbance at 230 nm (*A*_230_) as a function of rv. Peaks at ∼12, ∼13, and ∼15 mL correspond to trimeric assemblies of ***B***-***M***-***E*** with estimated molar masses of 129 kDa (***B-M-E***_20_), 155 kDa (***B-M-E***_40_), and 207 kDa (***B-M-E***_80_), respectively. (c) MALDI-TOF mass spectrum for 1 mg/mL ***B***-***M***-***E***_20_. (d) CD spectra, mean residue molar ellipticity ([θ]) versus wavelength (λ). Blue line: initial spectrum at 20 °C, orange line: spectrum after heating at 1 °C/min to 95 °C, and green line: spectrum after cooling back to 20 °C at 1 °C/min. Note: the blue and green curves almost overlap fully within the experimental error. (e) Mean residue molar ellipticity ([θ]) at λ = 222 nm plotted as a function of temperature during a heating ramp (1 °C/min). All CD measurements were performed using ***B***-***M***-***E***_20_ in Milli-Q water at 0.1 mg/mL.

Synthetic genes encoding ***B***-***M***-***E***_20_, ***B***-***M***-***E***_40_, and ***B***-***M***-***E***_80_ proteins were synthesized and cloned into a vector with a T7 promoter system. Proteins were expressed in *E. coli* and purified using IMAC, followed by SEC. Figure 2 summarizes the results for protein purification and characterization of ***B***-***M***-***E***_20_, ***B***-***M***-***E***_40_, and ***B***-***M***-***E***_80_. Additional data is shown in Figure S1–S3. SDS-PAGE analysis of the final purified proteins shows a single band (Figure 2a), and the proteins elute as a single peak in analytical SEC (Figure 2b). Peaks at ∼12, ∼13, and ∼15 mL retention volume (rv) correspond to trimeric assemblies of ***B***-***M***-***E*** with estimated molar masses of 129 kDa (***B-M-E***_20_), 155 kDa (***B-M-E***_40_), and 207 kDa (***B-M-E***_80_), respectively. To more precisely establish the mass of the purified polypeptides, we used MALDI-TOF mass spectrometry. Results for ***B***-***M***-***E***_20_ are shown in Figure 2c (results for ***B***-***M***-***E***_40_ and ***B***-***M***-***E***_80_ are shown in Figures S2b and S3b**)**. We find that the mass experimentally determined for ***B***-***M***-***E***_20_ (43 088.1 Da) matches the theoretically expected value (43 064.3 Da) within the error of the measurement, and the same holds for ***B***-***M***-***E***_40_ and ***B***-***M***-***E***_80_ as shown in Table 1.

**Table 1:** Theoretically and MALDI-TOF Measured Molecular Weights of ***B-M-E*** Proteins

protein	expected (Da)	MALDI-TOF (Da)
***B***-***M***-***E***_20_	43 064.3	43 088.1
***B***-***M***-***E***_40_	51 704.0	51 712.4
***B***-***M***-***E***_80_	68 983.4	69 007.6

Next, we investigated the protein secondary structure using CD spectroscopy. The ***M*** domain is exclusively α-helical,^39^ and if the experimental CD spectra are consistent with that of mainly a α-helical protein, we can have good confidence that the trimerization domain ***M*** is correctly folded within the full-length ***B***-***M***-***E***_20_. Results for the CD spectrum of ***B***-***M***-***E***_20_ are shown in Figure 2c and indeed show a spectrum consistent with a largely α-helical protein, confirming that the ***M*** domain is correctly folded within the full length of ***B***-***M***-***E***_20_. The ***M*** domain has previously been reported to be highly thermostable.^39^ We have also found this to be the case for the ***M*** domain in the context of our previously designed silica-coating proteins.^32^Figure 2d shows that, as expected, the same holds for the ***M*** domain in the gold-coating proteins: the spectra do not change when heating from 20 to 95 °C and cooling back to 20 °C (20 °C rev). Indeed, Figure 2e shows the mean residue molar ellipticity at 222 nm as a function of temperature and demonstrates that there is no sign of any thermal transition when heating from 20 to 95 °C.

### Coating Formation

**Figure 3:**
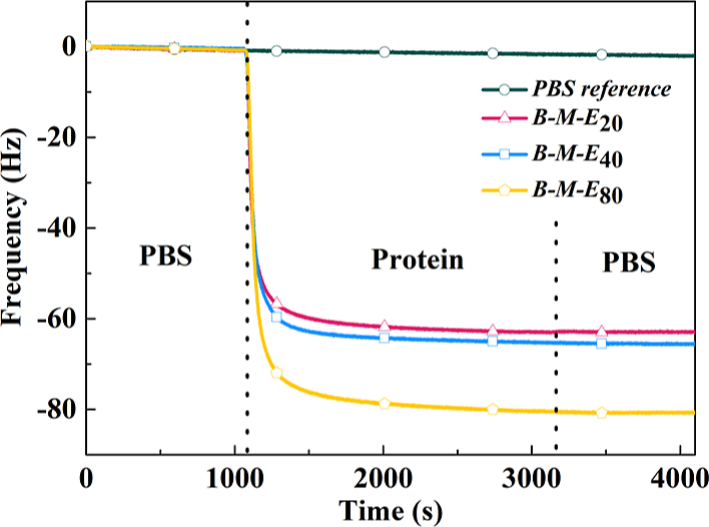
QCM-D results for coating formation on the gold surface of ***B***-***M***-***E***_20_, ***B***-***M***-***E***_40_, and ***B***-***M***-***E***_80_. The red line with a triangle, blue line with a square, and yellow line with a pentagon correspond to, respectively, ***B****-****M****-****E***_20_, ***B****-****M****-****E***_40_, and ***B****-****M****-****E***_80_. Blue-green line with a circle is the reference line (only flushing with PBS). QCM frequency shift (Hz) versus time (s). The first vertical dotted line corresponds to the injection of 10 μM respective ***B-M-E*** proteins. At the second vertical dotted line, we switch back to rinsing with PBS. All measurements were performed at a flow rate of 50 μL/min, and all samples for QCM-D were prepared in PBS buffer.

Next, we tested the formation of protein coating by the ***B***-***M***-***E*** proteins on gold surfaces. In our earlier work on silica-coating proteins,^32,35^ we found that ***B***-***E*** designs with only one silica-binding domain could still be rinsed off with high salt buffers or displaced by serum proteins. However, this was no longer the case for the ***B***-***M***-***E*** designs with multivalent surface anchorage. Here, we use QCM-D to investigate whether the new series of ***B***-***M***-***E*** triblocks, redesigned to bind to gold surfaces, form protein brushes on gold surfaces in PBS buffer. The QCM-D results for coating formation by ***B***-***M***-***E***_20_, ***B***-***M***-***E***_40_, and ***B***-***M***-***E***_80_ on gold surfaces are shown in Figure 3. Upon injecting the ***B***-***M***-***E*** protein solutions, the quartz crystal oscillation frequencies decrease, indicating protein layer formation, with the magnitude of the frequency drop being proportional to the length of the ***E*** block. According to the Sauerbrey equation,^57^ the frequency drop is only linearly related to adsorbed mass in QCM-D for adsorbed layers with low dissipation. Previously, we have found that the silica-binding ***B***-***M***-***E*** proteins form highly hydrated polypeptide brushes with strong dissipation.^32^ Rather than trying to fit our data with complicated models fully accounting for the dissipation, we here simply use the frequency drop as a qualitative measure for adsorbed mass.

Before injecting proteins, a flat baseline was obtained by flushing the gold sensor with PBS for 20 min. Then, the frequency signal decreased when proteins were injected into the QCM channels, which means proteins were binding to surfaces. Around 10 min after starting to inject the ***B***-***M***-***E*** proteins, the frequency shift signal saturates, indicating that no additional protein is bound. Next, QCM-D sensors were flushed with PBS. This did not lead to any observable change in the QCM signal, indicating that no washing off of any potentially weakly bound ***B***-***M***-***E*** proteins took place, nor did the PBS buffer displace bound ***B***-***M***-***E*** proteins. We therefore conclude that the trivalent binding of the GBP1 peptide is sufficiently strong.

To verify that the proteins coat the gold surface homogeneously, we performed AFM imaging of dried layers of the ***B***-***M***-***E*** proteins adsorbed on gold surfaces.

**Figure 4:**
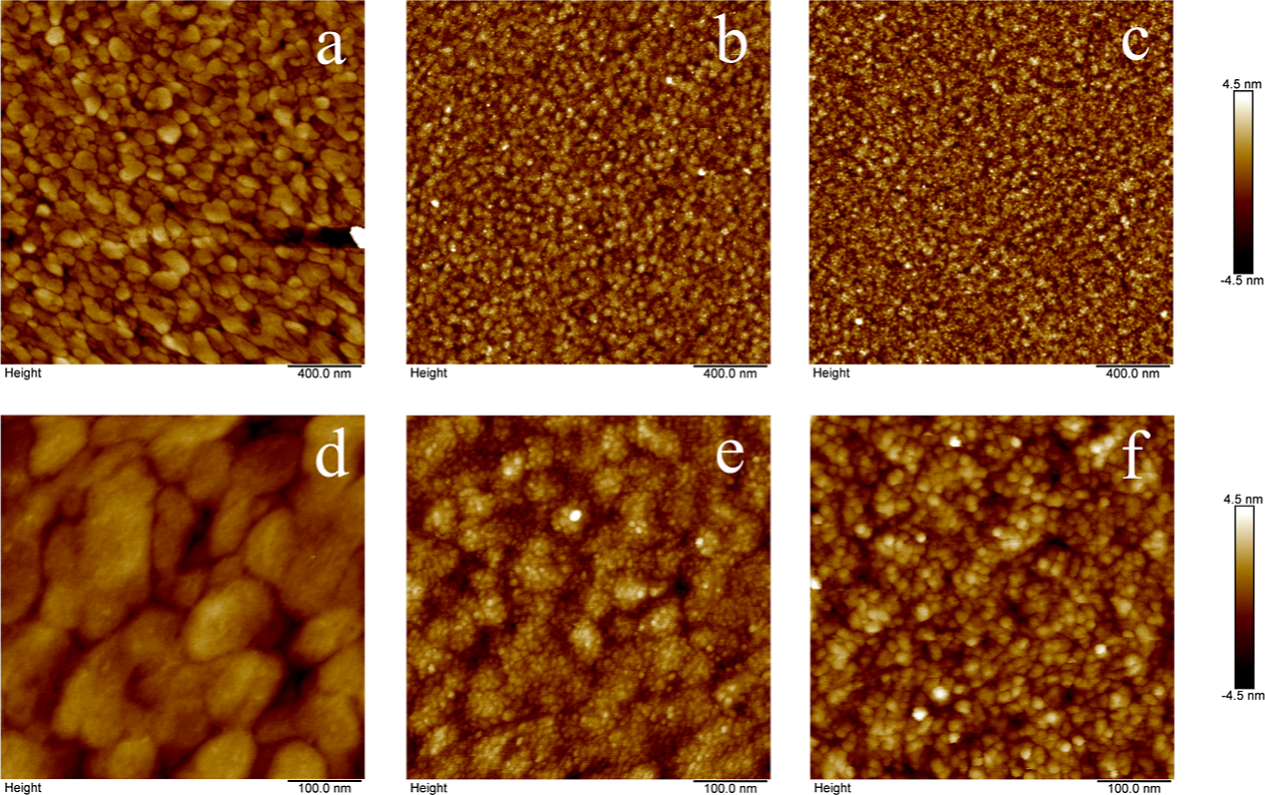
AFM images of bare gold surface and gold surface coated with ***B***-***M***-***E***_20_ with different concentrations and times. (a) 2 μm × 2 μm bare gold surface; (b) 2 μm × 2 μm gold surface, and 0.5 μM ***B***-***M***-***E***_20_ for 3 s; (c) 2 μm × 2 μm gold surface and 5 μM ***B***-***M***-***E***_20_ for 5 min; (d) 500 nm × 500 nm bare gold surface; (e) 500 nm × 500 nm gold surface and 0.5 μM ***B***-***M***-***E***_20_ for 3 s; and (f) 500 nm × 500 nm gold surface and 5 μM ***B***-***M***-***E***_20_ for 5 min. All measurements were performed in air. Gold sensor chips used are glass slides with a 45–50 nm gold thin film deposited onto an adhesion layer (Plasmetrix Technologies Inc, Canada).

More specifically, we want to verify that the ***B***-***M***-***E*** trimers adsorb as independent units and do not adsorb in clusters or as aggregates. This is best studied at conditions leading to lower coverage than the conditions used in the QCM-D coating formation experiments. Dried samples were imaged in air. Representative AFM images for ***B***-***M***-***E***_20_ are shown in Figure 4. The bare gold surface is a sputter-coated SPR sensor chip. The gold sensor surface topography is shown in Figure 4a,d. Images after incubation with either 0.5 μM ***B***-***M***-***E***_20_ for 3 s or 5 μM ***B***-***M***-*E*_20_ for 5 min are shown in Figure 4b,e and Figure 4c,f, respectively. These images clearly show that protein adsorption is extremely homogeneous: proteins did not adsorb in clusters, nor was there any sign of protein aggregates binding to the gold.

To confirm that the additional feature visible in the AFM images are indeed due to the adsorbed protein, we performed XPS measurements on samples identical to those used in AFM. XPS results are shown in Figure S3 and Table S4. These showed that only for the samples incubated with protein, and not for the bare surfaces, there are clear carbon and nitrogen signals, next to the Au signal that is present for all samples.

To determine the layer thickness of the ***B***-***M***-***E*** polypeptide brushes self-assembled onto gold surfaces, we used dynamic light scattering (DLS). As shown earlier for silica nanoparticles and the silica-coating ***B***-***M***-***E*** polypeptides,^32^ bridging interactions may lead to particle clustering at very low concentrations, but at higher concentrations, a fully saturated layer develops, leading to a small but measurable increase in the particle hydrodynamic diameter, from which we can estimate the layer thickness.

**Figure 5:**
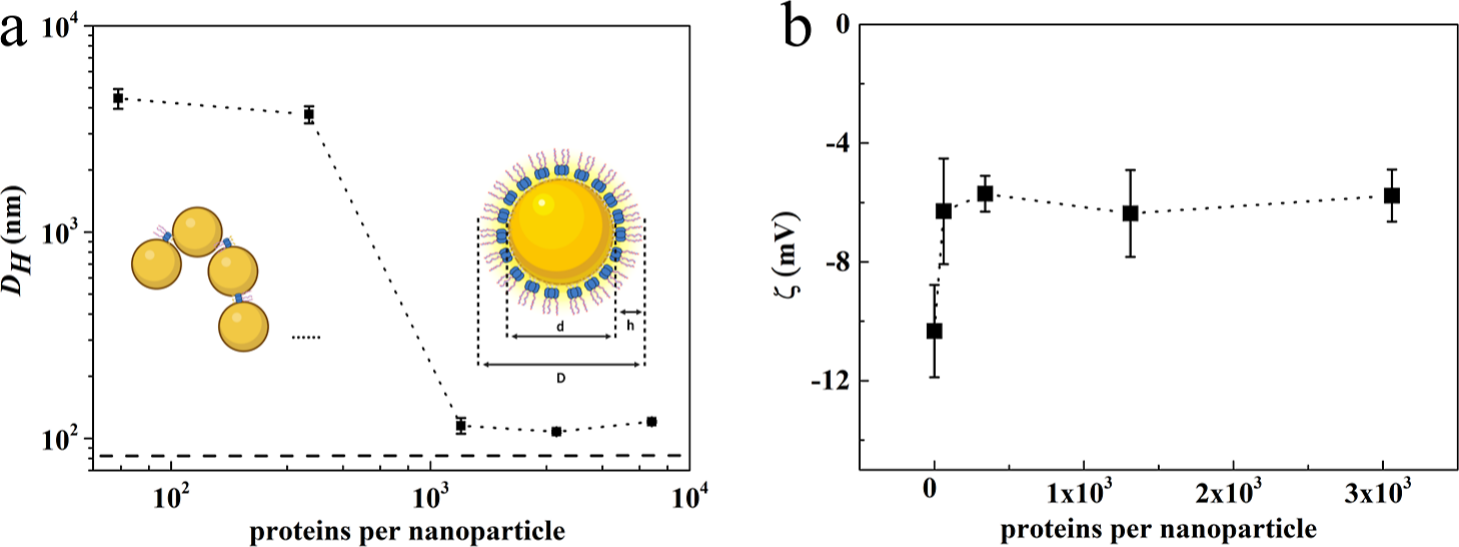
Interaction of ***B***-***M***-***E***_20_ triblocks with *d* = 83 nm diameter gold particles. (a) Effective hydrodynamic diameter (nm) of the particles as determined using DLS versus proteins per nanoparticle. The horizontal dashed line represents the diameter (83 nm) of the bare gold particles. The diameter of ***B***-***M***-***E***_20_-coated GNPs is *D* = 107 nm, suggesting a layer thickness *h* for the ***B***-***M***-***E***_20_ coating of *h* = (*D* – *d*)/2 = 12 nm. All DLS measurements are averages of 15 independent measurements. Insets are cartoons of GNPs being bridged by ***B***-***M***-***E*** proteins at low concentrations (left) and of fully coated GNPs at high concentrations (right). (b) Zeta potential ζ (mV) of GNPs coated with ***B***-***M***-***E***_20_ proteins, as a function of the bulk concentration of ***B***-***M***-***E***_20_.

We used GNPs with a hydrodynamic diameter *d* = 83 nm. Dilute suspensions of the GNPs were incubated for 5 min at different concentrations of the gold-binding ***B***-***M***-***E*** proteins. Results for DLS size measurements and zeta potential measurements for the case of ***B***-***M***-***E***_20_ are shown in Figure 5. Concentrations are expressed as the number of proteins per GNP, where we used the concentration of GNP in the original stock solution as specified by the manufacturer. Note that, due to mass balance, this is not the same as the actual coverage of protein on the GNP, which will be less due to the presence of proteins in solution, but which is expected to be in the range of 100–1000 proteins per GNP.

As shown in Figure 5a, at very low ***B***-***M***-***E***_20_ concentrations, DLS suggests significant bridging-induced GNP clustering similar to what was observed earlier for ***B***-***M***-***E*** proteins and silica nanoparticles. Bridging could be caused by interactions of the GNPs with any of the three domains of the ***B***-***M***-***E*** protein, as indicated in the inset of Figure 5a.

At high concentrations, the measured hydrodynamic diameters *D* stabilize at a value somewhat larger than the diameter *d* of the uncoated GNP. The diameter of ***B***-***M***-***E***_20_-coated gold nanoparticles is *D* = 107 nm, suggesting a layer thickness *h* for the ***B***-***M***-***E***_20_ coating of *h* = (*D* – *d*)/2 = 12 nm. Results for ***B***-***M***-***E***_40_ and ***B***-***M***-***E***_80_ were similar, although, as expected, the layer thickness increases with the length of the ***E*** block. These DLS data are shown in Figures S5 and S6.

To probe for any dependence of layer formation on GNP size, we also performed the DLS measurement for ***B***-***M***-***E***_20_ using smaller GNPs with a hydrodynamic diameter *d* = 42 nm (Figure S11). We find a very similar layer thickness of *h* = 13 nm. Due to the lower scattering of the smaller GNP, at high protein concentrations, one can now also observe a peak in the DLS signal due to the dissolved proteins. This peak corresponds to a hydrodynamic diameter *d* = 14 nm for the ***B***-***M***-***E*** trimer, which is of the same order of magnitude as the layer thicknesses we find.

Results for the zeta potential of GNPs and GNPs coated with ***B***-***M***-***E***_20_ proteins are shown in Figure 5b. We find that upon coating the GNPs with ***B***-***M***-***E***_20_, the zeta potential becomes somewhat less negative, changing from −10 mV for uncoated GNP to −6 mV for coated GNP.

### Coating Functionality

Our aim is to render the gold surfaces antifouling and, at a later stage, to make the coating layers functional, for example, for use in biosensor surfaces.^10,58^ Therefore, we next tested the extent to which the ***B-M-E*** coatings can prevent non-specific protein adsorption. In our previous study,^32^ we found that the silica-binding ***B***-***M***-***E*** protein performed well against 1 mg/mL BSA. Here, we investigate biofouling for ***B***-***M***-***E*** layers assembled on gold surfaces against diluted HS (1% HS and 10% HS, in PBS, pH 7.4).

**Figure 6:**
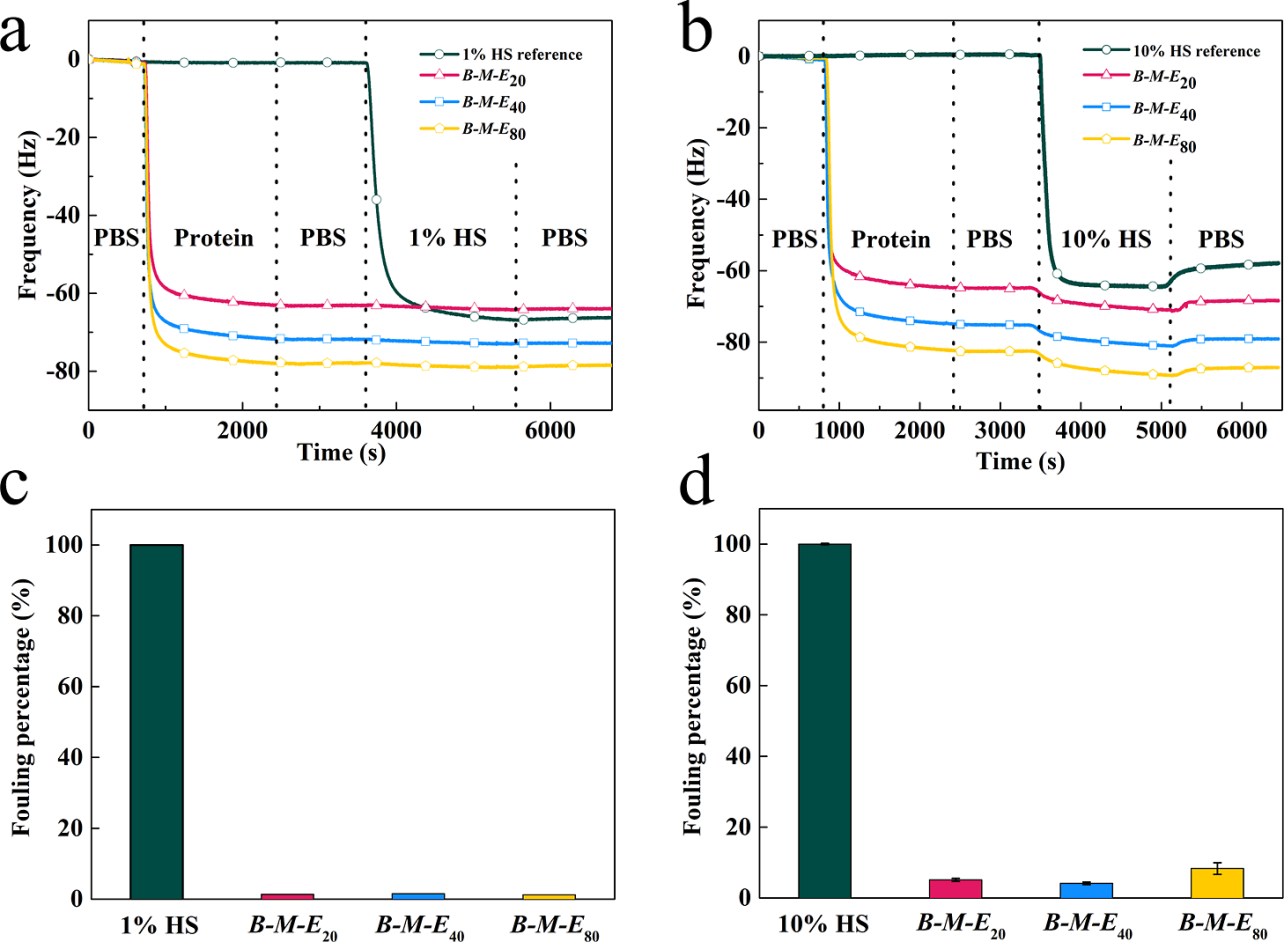
QCM-D assay of ***B-M-E*** antifouling activity. (a,b) QCM frequency shift (Hz) versus time *t* (s) after start of injection of 10 μM ***B-M-E*** protein. The red line with triangle, blue line with square, and yellow line with pentagon represent ***B***-***M***-***E***_20_, ***B***-***M***-***E***_40_, and ***B***-***M***-***E***_80_ binding to the gold surface, respectively. First, a flat baseline was obtained by flushing with PBS; 10 μM ***B-M-E*** protein was injected; PBS was injected again; next, (a) 1% HS or (b) 10% HS was injected; finally, PBS was injected again. The reference channel is shown in a blue-green line with a circle, the same as other measurements, but without the ***B-M-E*** coating. (c,d) Quantitative analysis of HS fouling on bare gold surfaces and surfaces with the ***B-M-E*** coating. Frequency change between the timepoints just before the injection of HS and the end of the final rinse with PBS, normalized by the frequency change for the reference case (no coating, bare gold surfaces). (c) 1% HS and (d) 10% HS.

Representative QCM-D results of antifouling tests with diluted HS are shown in Figure 6. As shown, first an adsorbed ***B***-***M***-***E*** layer was formed and extensively rinsed with PBS. Next, the protein foulant was injected to the QCM-D channels and finally rinsed again with PBS. Results for 1% HS and 10% HS are shown in Figure 6a,b, respectively. As a control, an uncoated gold sensor was also included. For this control case, upon injecting the protein foulants, there is a significant drop in the QCM-D frequency, indicating significant fouling, as expected, and in line with ample literature data on the adsorption of serum proteins onto negatively charged inorganic surfaces.^59^

The signal for serum proteins adsorbing to the bare gold sensors is smaller than that for the coating proteins adsorbing to the gold sensors since the ***B***-***M***-***E*** proteins are trimers of rather high molar mass and they adsorb not as rigid flat layers but as thick, hydrated polymer brushes, as we discuss in more detail in further paragraphs.

After rinsing, for the serum proteins adsorbed on the bare gold sensor, part of the fouling can be removed again, but most appears to have adsorbed irreversibly. In contrast, for the sensors first coated with the three gold-binding ***B***-***M***-***E*** proteins, hardly any fouling can be detected for the case of 1% HS (Figure 6a), whereas some (irreversible) fouling is observed for 10% HS (Figure 6b). A more quantitative analysis is shown in Figure 6c (for 1% HS) and Figure 6d (for 10% HS), where we plot the frequency change measured in QCM-D between the timepoints just before the injection of the protein foulant and the end of the final rinse with PBS, normalized by the frequency change for the reference case (no coating, bare gold surface).

At a fixed grafting density, for polymer brushes, the typical expectation is that antifouling increases with brush length. For our case of self-assembled polypeptide brushes (with variable grafting density), Figure 6d shows that the antifouling performance decreases somewhat for the longest brushes. This possibly points to a lower grafting density of these brushes, as will be discussed later. Additional data for antifouling at high concentrations of pure BSA is shown in Figure S7. For this case, hardly any fouling can be detected.

The sequence of the zwitterionic ELP block ***E***^***Z***^ used here is different from the serine-containing ELP block ***E***^***S***^ used for the silica-binding versions of the ***B***-***M***-***E*** protein studied before.^32^ To check whether the change to zwitterionic ELP ***E*** blocks indeed improves the antifouling performance, we also constructed the gold-binding ***B***-***M***-***E***_20_ protein with ***E*** = ***E***^***S***^ as the antifouling block. The antifouling performance of ***B***-***M***-***E***^***S***^_20_ and ***B***-***M***-***E****^Z^*_20_ layers adsorbed on gold against 10% HS is compared, and the results are shown in Figure S8. We find that the zwitterionic ELPs indeed offer a better antifouling performance, as hypothesized.

It is well known that protein adsorption from HS onto solid surfaces has complex kinetics with faster adsorbing components with low affinity gradually being replaced by more slowly adsorbing species with high affinity (Vroman effect^60,61^). Also, for the incubation times with the coating proteins used in Figure 6, coating formation had stabilized almost, but not completely yet. Therefore, for the case of ***B***-***M***-***E***_40_, we tested longer incubation times with coating proteins, as well as longer incubation times with 10% HS (Figures S9 and S10**)**. Results suggest that longer incubation times with the coating proteins, as expected, lead to a QCM signal that eventually becomes stable and remains stable after flushing with PBS (Figure S9). After longer incubation with 10% HS, we observe for the uncoated gold surface (Figure S10) but not for the coated gold surface (Figure S9) that during the final PBS rinse, there is somewhat more displacement than observed for shorter incubation times with 10% HS.

While a direct experimental determination of the orientation of the adsorbed ***B***-***M***-***E*** trimers is difficult, there are strong indirect indications that the binding blocks ***B*** are facing the side of the gold surface and the ***E*** blocks are facing the solution side and that the adsorbed layer is a monolayer as designed. We have pointed these out before; for the case of the silica-binding versions of the ***B***-***M***-***E*** proteins,^32^ the adsorbed layer thicknesses are of the order of the solution hydrodynamic sizes, and any solution side facing ***B*** blocks would for sure have compromised the antifouling behavior, which we do not observe.

In this work, we have relied on a non-thiol-containing gold-binding peptide sequence for surface anchoring to gold. A complementary and frequently used approach is to use thiol groups, for example, in cysteine residues, to strongly anchor molecules to gold surfaces.^49,62^ This chemistry has also been successfully employed to assemble antifouling (peptide) layers on gold surfaces.^50^ Cysteines could potentially be engineered into the binding blocks ***B***, but we have shown that even without cysteines, the trivalent gold-binding peptides offer sufficiently strong binding. Moreover, an advantage of using gold-binding peptides may be that binding is not influenced by the presence of reducing agents.

The main advantage, however, is that the modular ***B***-***M***-***E*** triblocks offer a multi-material solution that can be used to modify multiple materials in an identical manner. Indeed, a wide range of sequences for solid-binding peptides are available. For example, sequences for metal-binding peptides,^25,63^ mineral-binding peptides,^27,28^ plastic-binding peptides,^29,30^ and even for semiconductor-binding peptides.^31^ The potential for analogous ***B***-***M***-***E*** design-based protein coatings is therefore large.

In the literature on antifouling polymer brushes, it is generally reported that the degree of antifouling, at a given grafting density, increases with the length of the brush polymers.^64^ Somewhat to our surprise, we found no difference in the degree of antifouling for the ***B***-***M***-***E*** proteins with different lengths of the ***E*** block. In particular, we found that ***B***-***M***-***E***_20_, with the shortest antifouling block, works just as well as the ones with longer ***E*** blocks, ***B***-***M***-***E***_40_ and ***B***-***M***-***E***_80_.

Quite likely, the brush density of the self-assembled brushes arises from a competition between the binding blocks ***B***, which favor a brush density that is as high as possible. If the surface is not saturated with binding blocks, and the lateral brush pressure of antifouling blocks ***E*** favors a density as low as possible. Possibly, in the present case, this competition leads to significantly higher brush densities for the ***B***-***M***-***E***_20_ polymer, such that antifouling properties are maintained, even though the length of the antifouling block is short.

## Conclusions

By changing the sequences of the binding domain (***B***) and antifouling domains (***E***) of our original silica-coating triblock protein,^32^ we have demonstrated here that the ***B***-***M***-***E*** design that allows the formation of highly stable protein coatings is highly modular. For the current gold-binding peptides as the binding domain, excellent antifouling could be obtained using a zwitterionic ***E*** domain, ***E*** = ***E***^***Z***^.

It would be interesting to test whether even shorter antifouling blocks still lead to acceptable results. This would be highly relevant for the particular case of gold surfaces since especially for sensing schemes relying on local surface plasmon resonance, any coating needs to be as thin as possible.

Our current ***B-M-E*** designs provide excellent antifouling against 1% HS on gold surfaces. In at least some sensing applications, a 100-fold dilution with buffer may be acceptable or desired, but a much broader range of applications would be possible with an increased antifouling against, for example, 10% HS. This very likely requires coatings with a further increased surface density of more hydrophilic ***E*** blocks. This could possibly be achieved through a further optimization of both ***M*** and ***E*** blocks, and work toward this is ongoing.

Finally, many applications will involve functionalization of the ***E*** blocks, e.g., with capture molecules for biosensing applications. Especially, if the functional groups to be added are proteins or peptides, the protein nature of ***B-M-E*** is a great advantage, allowing for either direct inclusion of the functional domains in fusion designs or indirect attachment via one of the many available immobilization tags.

## Supporting Information Available

Primers, DNA sequences, amino acid sequences, and additional SDS-PAGE, MALDI-TOF, DLS, QCM, and XPS data (PDF)
