# Supplementary material for: Modular Design for Proteins Assembling into Antifouling Coatings: Case of Gold Surfaces
Source: Langmuir. 2023 Jun 27;39(27):9290–9. doi: 10.1021/acs.langmuir.3c00389 (PMC10339784; doi:10.1021/acs.langmuir.3c00389)
Supplement: Supplementary file 1 — la3c00389_si_001.pdf [file la3c00389_si_001.pdf]

Supporting Information

Modular Design for Proteins Assembling into Antifouling Coatings: Case of Gold Surfaces

Chuanbao Zheng,[1,2] Nicolò Alvisi,[1] Robbert Jan de Haas,[1] Zhisen Zhang,[3] Han Zuilhof,[2,4] and Renko de Vries[1]*

[1]Physical Chemistry and Soft Matter, Wageningen University & Research, Stippeneng 4, 6708 WE Wageningen, The Netherlands

[2]Laboratory of Organic Chemistry, Wageningen University & Research, Stippeneng 4, 6708 WE Wageningen, The Netherlands

[3] Research Institute for Biomimetics and Soft Matter, Fujian Provincial Key Laboratory for Soft Functional Materials Research, Department of Physics, Xiamen University, 361005 Xiamen, China

[4] School of Pharmaceutical Sciences and Technology, Tianjin University, 92 Weijin Road, Tianjin, 300072, China.

E-mail: renko.devries@wur.nl

Contents

Table S1. Primers used to obtain gene for H_6-$\boldsymbol{B}^{RT}$-$\boldsymbol{E}^{S}_{3}$-$\boldsymbol{M}^{HR00C\_3\_2}$.

Table S2. DNA sequences of all constructs.

Table S3. Amino acid sequences of all constructs.

Table S4. XPS results of different compounds percentage on gold surfaces before ***B-M-E₂₀*** coating and after coating.

Figure S1. Expression, purification results of constructs $\boldsymbol{B\text{-}M\text{-}E_{20}}$.

Figure S2. Expression, purification and MALDI-TOF results of constructs $\boldsymbol{B\text{-}M\text{-}E_{40}}$.

Figure S3. Expression, purification and MALDI-TOF results of constructs $\boldsymbol{B\text{-}M\text{-}E_{80}}$.

Figure S4. XPS results of different compounds on gold surfaces before $\boldsymbol{B\text{-}M\text{-}E_{20}}$ coating and after coating.

Figure S5. DLS results of constructs $\boldsymbol{B\text{-}M\text{-}E_{40}}$.

Figure S6. DLS results of constructs $\boldsymbol{B\text{-}M\text{-}E_{80}}$.

Figure S7. QCM-D data of ***B-M-E*** proteins against BSA.

Figure S8. ELPs sequence comparison between serine or charged amino acid.

Figure S9. QCM-D data of $\boldsymbol{B\text{-}M\text{-}E}_{40}$ proteins with longer incubation time against 10% HS.

Figure S10. QCM-D data of 10% HS binds to gold surface.

Figure S11. Interaction of $\boldsymbol{B\text{-}M\text{-}E}_{20}$ triblocks with d = 42 nm diameter gold particles.

Table S1. Primers used for obtaining gene for H_6-$\boldsymbol{B}^{RT}$-$\boldsymbol{E}^{S}_{3}$-$\boldsymbol{M}^{HR00C\_3\_2}$ from plasmid in reference.[1]

Primer	Sequences (5′ to 3′)
Primer-forward	AGGAAGTCGTAGCTGAGATGATC
Primer-reverse	CTCGATCCCATATGTACTCCTCC

Table S2. DNA sequences of all constructs.

The DNA sequences for the constructs H_6-$\boldsymbol{B}^{GBP1}$-$\boldsymbol{E}^{S}_{3}$-$\boldsymbol{M}^{HR00C\_3\_2}$-$\boldsymbol{E}^{Z}_{20}$, H_6-$\boldsymbol{B}^{GBP1}$-$\boldsymbol{E}^{S}_{3}$-$\boldsymbol{M}^{HR00C\_3\_2}$-$\boldsymbol{E}^{Z}_{40}$ and H_6-$\boldsymbol{B}^{GBP1}$-$\boldsymbol{E}^{S}_{3}$-$\boldsymbol{M}^{HR00C\_3\_2}$-$\boldsymbol{E}^{Z}_{80}$.

Construct name	DNA sequence
$\boldsymbol{B}^{GBP1}$	ATGCACGGGAAAACACAAGCTACTTCGGGAACCATCCAGAGC
$\boldsymbol{M}^{HR00C\_3\_2}$	ATGATCGAGGAAGTCGTAGCTGAGATGATCGACATCCTGGCGGAGTCCAGCAAGAAAAGCATCGAGGAATTAGCGCGTGCTGCCGATAACAAGACGACCGAGAAGGCCGTGGCCGAAGCGATTGAGGAAATTGCCCGCCTGGCGACCGCAGCGATTCAGCTGATCGAGGCATTAGCCAAGAATTTGGCGTCTGAGGAATTTATGGCCCGTGCAATTTCCGCCATCGCTGAATTGGCAAAGAAGGCAATTGAAGCAATCTATCGCTTGGCTGACAATCACACCACGGACACCTTCATGGCACGCGCCATCGCGGCGATCGCGAACCTCGCCGTTACTGCAATCTTGGCTATCGCCGCATTAGCATCGAATCATACAACCGAGGAATTCATGGCGCGTGCGATTTCCGCTATTGCAGAACTCGCGAAGAAAGCCATCGAAGCCATTTATCGCCTGGCAGATAACCATACCACGGATAAATTTATGGCGGCTGCTATCGAGGCCATCGCACTGCTTGCCACGCTGGCGATTCTGGCCATTGCCCTGCTTGC

	GAGTAATCACACAACTGAGGAATTCATGGCCAAAGCCAT<br>TAGTGCGATTGCGGAGTTGGCGAAGAAGGCGATCGAAG<br>CTATTTACCGTTTGGCGGATAACCACACAAGTCCAACCTA<br>TATCGAGAAGGCTATTGAGGCAATTGAGAAGATTGCGCG<br>CAAAGCGATTAAGGCCATTGAAATGCTGGCCAAGAACAT<br>CACCACGGAAGAATACAAAGAGAAAGCAAAATCGGCGA<br>TTGATGAAATCCGGGAAAAGGCGAAGGAAGCGATCAAA<br>CGTTTAGAAGACAATCGTACC
**H$_6$-B^{GBP1}-E^{S}_{3}-$M^{HR00C\_3\_2}$-E^{Z}_{20}**	ATGGGATCGAGCCATCACCATCACCATCATTCGAGCGGA<br>ATGCACGGGAAAACACAAGCTACTTCGGGAACCATCCA<br>GAGCGGCTCTGGCGTCCCAGGTTCCGGTGTACCTGGATC<br>TGGCGTACCGATGATCGAGGAAGTCGTAGCTGAGATGAT<br>CGACATCCTGGCGGAGTCCAGCAAGAAAAGCATCGAGG<br>AATTAGCGCGTGCTGCCGATAACAAGACGACCGAGAAG<br>GCCGTGGCCGAAGCGATTGAGGAAATTGCCCGCCTGGC<br>GACCGCAGCGATTCAGCTGATCGAGGCATTAGCCAAGAA<br>TTTGGCGTCTGAGGAATTTATGGCCCGTGCAATTTCCGCC<br>ATCGCTGAATTGGCAAAGAAGGCAATTGAAGCAATCTAT<br>CGCTTGGCTGACAATCACACCACGGACACCTTCATGGCA<br>CGCGCCATCGCGGCGATCGCGAACCTCGCCGTTACTGCA<br>ATCTTGGCTATCGCCGCATTAGCATCGAATCATACAACCG

	AGGAATTCATGGCGCGTGCGATTTCCGCTATTGCAGAAC<br>TCGCGAAGAAAGCCATCGAAGCCATTTATCGCCTGGCAG<br>ATAACCATACCACGGATAAATTTATGGCGGCTGCTATCGA<br>GGCCATCGCACTGCTTGCCACGCTGGCGATTCTGGCCAT<br>TGCCCTGCTTGCGAGTAATCACACAACTGAGGAATTCAT<br>GGCCAAAGCCATTAGTGCGATTGCGGAGTTGGCGAAGA<br>AGGCGATCGAAGCTATTTACCGTTTGGCGGATAACCACA<br>CAAGTCCAACCTATATCGAGAAGGCTATTGAGGCAATTG<br>AGAAGATTGCGCGCAAAGCGATTAAGGCCATTGAAATGC<br>TGGCCAAGAACATCACCACGGAAGAATACAAAGAGAAA<br>GCAAAATCGGCGATTGATGAAATCCGGGAAAAGGCGAA<br>GGAAGCGATCAAACGTTTAGAAGACAATCGTACCGGTG<br>ATGGTGTGCCGGGTAAGGGAGTTCCAGGTGACGGCGTTC<br>CTGGTAAAGGTGTTCCGGGGGATGGTGTACCGGGAAAG<br>GGTGTCCCGGGGGACGGAGTTCCGGGAAAAGGCGTTCC<br>TGGCGATGGCGTGCCTGGGAAGGGCGTACCGGGTGACG<br>GTGTTCCGGGCAAAGGGGTTCCAGGCGACGGAGTCCCA<br>GGGAAAGGTGTGCCTGGAGATGGCGTCCCAGGCAAAGG<br>AGTCCCGGGTGATGGAGTGCCAGGGAAGGGCGTCCCTG<br>GTGATGGCGTCCCAGGTAAGGGCGTCCCAGGC

**H$_6$-*B*^{GBP1}-*E*$^S{}_3$-*M*$^{HR00C\_3\_2}$-*E*$^Z{}_{40}$**	ATGGGATCGAGCCATCACCATCACCATCATTCGAGCGGA<br>ATGCACGGGAAAACACAAGCTACTTCGGGAACCATCCA<br>GAGCGGCTCTGGCGTCCCAGGTTCCGGTGTACCTGGATC<br>TGGCGTACCGATGATCGAGGAAGTCGTAGCTGAGATGAT<br>CGACATCCTGGCGGAGTCCAGCAAGAAAAGCATCGAGG<br>AATTAGCGCGTGCTGCCGATAACAAGACGACCGAGAAG<br>GCCGTGGCCGAAGCGATTGAGGAAATTGCCCGCCTGGC<br>GACCGCAGCGATTCAGCTGATCGAGGCATTAGCCAAGAA<br>TTTGGCGTCTGAGGAATTTATGGCCCGTGCAATTTCCGCC<br>ATCGCTGAATTGGCAAAGAAGGCAATTGAAGCAATCTAT<br>CGCTTGGCTGACAATCACACCACGGACACCTTCATGGCA<br>CGCGCCATCGCGGCGATCGCGAACCTCGCCGTTACTGCA<br>ATCTTGGCTATCGCCGCATTAGCATCGAATCATACAACCG<br>AGGAATTCATGGCGCGTGCGATTTCCGCTATTGCAGAAC<br>TCGCGAAGAAAGCCATCGAAGCCATTTATCGCCTGGCAG<br>ATAACCATACCACGGATAAATTTATGGCGGCTGCTATCGA<br>GGCCATCGCACTGCTTGCCACGCTGGCGATTCTGGCCAT<br>TGCCCTGCTTGCGAGTAATCACACAACTGAGGAATTCAT<br>GGCCAAAGCCATTAGTGCGATTGCGGAGTTGGCGAAGA<br>AGGCGATCGAAGCTATTTACCGTTTGGCGGATAACCACA<br>CAAGTCCAACCTATATCGAGAAGGCTATTGAGGCAATTG

	AGAAGATTGCGCGCAAAGCGATTAAGGCCATTGAAATGC<br>TGGCCAAGAACATCACCACGGAAGAATACAAAGAGAAA<br>GCAAAATCGGCGATTGATGAAATCCGGGAAAAGGCGAA<br>GGAAGCGATCAAACGTTTAGAAGACAATCGTACCGGTG<br>ATGGTGTGCCGGGTAAGGGAGTTCCAGGTGACGGCGTTC<br>CTGGTAAAGGTGTTCCGGGGGATGGTGTACCGGGAAAG<br>GGTGTCCCGGGGGACGGAGTTCCGGGAAAAGGCGTTCC<br>TGGCGATGGCGTGCCTGGGAAGGGCGTACCGGGTGACG<br>GTGTTCCGGGCAAAGGGGTTCCAGGCGACGGAGTCCCA<br>GGGAAAGGTGTGCCTGGAGATGGCGTCCCAGGCAAAGG<br>AGTCCCGGGTGATGGAGTGCCAGGGAAGGGCGTCCCTG<br>GTGATGGCGTCCCAGGTAAGGGCGTCCCAGGTGATGGTG<br>TGCCGGGTAAGGGAGTTCCAGGTGACGGCGTTCCTGGTA<br>AAGGTGTTCCGGGGGATGGTGTACCGGGAAAGGGTGTC<br>CCGGGGGACGGAGTTCCGGGAAAAGGCGTTCCTGGCGA<br>TGGCGTGCCTGGGAAGGGCGTACCGGGTGACGGTGTTC<br>CGGGCAAAGGGGTTCCAGGCGACGGAGTCCCAGGGAA<br>AGGTGTGCCTGGAGATGGCGTCCCAGGCAAAGGAGTCC<br>CGGGTGATGGAGTGCCAGGGAAGGGCGTCCCTGGTGAT<br>GGCGTCCCAGGTAAGGGCGTCCCAGGC

**H_6-*B*^{GBP1}-*E*$^{S}_{3}$-*M*$^{HR00C\_3\_2}$-*E*$^{Z}_{80}$**	ATGGGATCGAGCCATCACCATCACCATCATTCGAGCGGA<br>ATGCACGGGAAAACACAAGCTACTTCGGGAACCATCCA<br>GAGCGGCTCTGGCGTCCCAGGTTCCGGTGTACCTGGATC<br>TGGCGTACCGATGATCGAGGAAGTCGTAGCTGAGATGAT<br>CGACATCCTGGCGGAGTCCAGCAAGAAAAGCATCGAGG<br>AATTAGCGCGTGCTGCCGATAACAAGACGACCGAGAAG<br>GCCGTGGCCGAAGCGATTGAGGAAATTGCCCGCCTGGC<br>GACCGCAGCGATTCAGCTGATCGAGGCATTAGCCAAGAA<br>TTTGGCGTCTGAGGAATTTATGGCCCGTGCAATTTCCGCC<br>ATCGCTGAATTGGCAAAGAAGGCAATTGAAGCAATCTAT<br>CGCTTGGCTGACAATCACACCACGGACACCTTCATGGCA<br>CGCGCCATCGCGGCGATCGCGAACCTCGCCGTTACTGCA<br>ATCTTGGCTATCGCCGCATTAGCATCGAATCATACAACCG<br>AGGAATTCATGGCGCGTGCGATTTCCGCTATTGCAGAAC<br>TCGCGAAGAAAGCCATCGAAGCCATTTATCGCCTGGCAG<br>ATAACCATACCACGGATAAATTTATGGCGGCTGCTATCGA<br>GGCCATCGCACTGCTTGCCACGCTGGCGATTCTGGCCAT<br>TGCCCTGCTTGCGAGTAATCACACAACTGAGGAATTCAT<br>GGCCAAAGCCATTAGTGCGATTGCGGAGTTGGCGAAGA<br>AGGCGATCGAAGCTATTTACCGTTTGGCGGATAACCACA<br>CAAGTCCAACCTATATCGAGAAGGCTATTGAGGCAATTG

	AGAAGATTGCGCGCAAAGCGATTAAGGCCATTGAAATGC<br>TGGCCAAGAACATCACCACGGAAGAATACAAAGAGAAA<br>GCAAAATCGGCGATTGATGAAATCCGGGAAAAGGCGAA<br>GGAAGCGATCAAACGTTTAGAAGACAATCGTACCGGTG<br>ATGGTGTGCCGGGTAAGGGAGTTCCAGGTGACGGCGTTC<br>CTGGTAAAGGTGTTCCGGGGGATGGTGTACCGGGAAAG<br>GGTGTCCCGGGGGACGGAGTTCCGGGAAAAGGCGTTCC<br>TGGCGATGGCGTGCCTGGGAAGGGCGTACCGGGTGACG<br>GTGTTCCGGGCAAAGGGGTTCCAGGCGACGGAGTCCCA<br>GGGAAAGGTGTGCCTGGAGATGGCGTCCCAGGCAAAGG<br>AGTCCCGGGTGATGGAGTGCCAGGGAAGGGCGTCCCTG<br>GTGATGGCGTCCCAGGTAAGGGCGTCCCAGGTGATGGTG<br>TGCCGGGTAAGGGAGTTCCAGGTGACGGCGTTCCTGGTA<br>AAGGTGTTCCGGGGGATGGTGTACCGGGAAAGGGTGTC<br>CCGGGGGACGGAGTTCCGGGAAAAGGCGTTCCTGGCGA<br>TGGCGTGCCTGGGAAGGGCGTACCGGGTGACGGTGTTC<br>CGGGCAAAGGGGTTCCAGGCGACGGAGTCCCAGGGAA<br>AGGTGTGCCTGGAGATGGCGTCCCAGGCAAAGGAGTCC<br>CGGGTGATGGAGTGCCAGGGAAGGGCGTCCCTGGTGAT<br>GGCGTCCCAGGTAAGGGCGTCCCAGGTGATGGTGTGCC<br>GGGTAAGGGAGTTCCAGGTGACGGCGTTCCTGGTAAAG

	GTGTTCCGGGGGATGGTGTACCGGGAAAGGGTGTCCCG<br>GGGGACGGAGTTCCGGGAAAAGGCGTTCCTGGCGATGG<br>CGTGCCTGGGAAGGGCGTACCGGGTGACGGTGTTCCGG<br>GCAAAGGGGTTCCAGGCGACGGAGTCCCAGGGAAAGGT<br>GTGCCTGGAGATGGCGTCCCAGGCAAAGGAGTCCCGGG<br>TGATGGAGTGCCAGGGAAGGGCGTCCCTGGTGATGGCG<br>TCCCAGGTAAGGGCGTCCCAGGTGATGGTGTGCCGGGTA<br>AGGGAGTTCCAGGTGACGGCGTTCCTGGTAAAGGTGTTC<br>CGGGGGATGGTGTACCGGGAAAGGGTGTCCCGGGGGAC<br>GGAGTTCCGGGAAAAGGCGTTCCTGGCGATGGCGTGCC<br>TGGGAAGGGCGTACCGGGTGACGGTGTTCCGGGCAAAG<br>GGGTTCCAGGCGACGGAGTCCCAGGGAAAGGTGTGCCT<br>GGAGATGGCGTCCCAGGCAAAGGAGTCCCGGGTGATGG<br>AGTGCCAGGGAAGGGCGTCCCTGGTGATGGCGTCCCAG<br>GTAAGGGCGTCCCAGGC

Table S3. Amino acid sequences of all constructs.

The amino acid sequences for the constructs H_6-$\boldsymbol{B}^{GBP1}$-$\boldsymbol{E}^{S}_{3}$-$\boldsymbol{M}^{HR00C\_3\_2}$-$\boldsymbol{E}^{Z}_{20}$, H_6-$\boldsymbol{B}^{GBP1}$-$\boldsymbol{E}^{S}_{3}$-$\boldsymbol{M}^{HR00C\_3\_2}$-$\boldsymbol{E}^{Z}_{40}$ and H_6-$\boldsymbol{B}^{GBP1}$-$\boldsymbol{E}^{S}_{3}$-$\boldsymbol{M}^{HR00C\_3\_2}$-$\boldsymbol{E}^{Z}_{80}$.

Construct name	Amino acid sequence
B(GBP1)	MHGKTQATSGTIQS
$\boldsymbol{M}^{HR00C\_3\_2}$	MIEEVVAEMIDILAESSKKSIEELARAADNKTTEKAVAEAIE EIARLATAAIQLIEALAKNLASEEFMARAISAIAELAKKAIE AIYRLADNHTTDTFMARAIAAIANLAVTAILAIAALASNHT TEEFMARAISAIAELAKKAIEAIYRLADNHTTDKFMAAAIE AIALLATLAILAIALLASNHTTEEFMAKAISAIAELAKKAIE AIYRLADNHTSPTYIEKAIEAIEKIARKAIKAIEMLAKNITTE EYKEKAKSAIDEIREKAKEAIKRLEDNRT
**H_6-$\boldsymbol{B}^{GBP1}$-$\boldsymbol{E}^{S}_{3}$-$\boldsymbol{M}^{HR00C\_3\_2}$-$\boldsymbol{E}^{Z}_{20}$**	MGSSHHHHHHSSGMHGKTQATSGTIQSGSGVPGSGVPGSG VPMIEEVVAEMIDILAESSKKSIEELARAADNKTTEKAVAE AIEEIARLATAAIQLIEALAKNLASEEFMARAISAIAELAKK AIEAIYRLADNHTTDTFMARAIAAIANLAVTAILAIAALASN HTTEEFMARAISAIAELAKKAIEAIYRLADNHTTDKFMAA AIEAIALLATLAILAIALLASNHTTEEFMAKAISAIAELAKK AIEAIYRLADNHTSPTYIEKAIEAIEKIARKAIKAIEMLAKNI TTEEYKEKAKSAIDEIREKAKEAIKRLEDNRTGDGVPGKG

	VPGDGVPGKGVPGDGVPGKGVPGDGVPGKGVPGDGVPG<br>KGVPGDGVPGKGVPGDGVPGKGVPGDGVPGKGVPGDGV<br>PGKGVPGDGVPGKGVPG
**H_6-*B*^{GBP1}-*E*$^{S}_{3}$-*M*$^{HR00C\_3\_2}$-*E*$^{Z}_{40}$**	MGSSHHHHHHSSGMHGKTQATSGTIQSGSGVPGSGVPGSG<br>VPMIEEVVAEMIDILAESSKKSIEELARAADNKTTEKAVAE<br>AIEEIARLATAAIQLIEALAKNLASEEFMARAISAIAELAKK<br>AIEAIYRLADNHTTDTFMARAIAAIANLAVTAILAIAALASN<br>HTTEEFMARAISAIAELAKKAIEAIYRLADNHTTDKFMAA<br>AIEAIALLATLAILAIALLASNHTTEEFMAKAISAIAELAKK<br>AIEAIYRLADNHTSPTYIEKAIEAIEKIARKAIKAIEMLAKNI<br>TTEEYKEKAKSAIDEIREKAKEAIKRLEDNRTGDGVPGKG<br>VPGDGVPGKGVPGDGVPGKGVPGDGVPGKGVPGDGVPG<br>KGVPGDGVPGKGVPGDGVPGKGVPGDGVPGKGVPGDGV<br>PGKGVPGDGVPGKGVPGDGVPGKGVPGDGVPGKGVPGD<br>GVPGKGVPGDGVPGKGVPGDGVPGKGVPGDGVPGKGVP<br>GDGVPGKGVPGDGVPGKGVPGDGVPGKGVPGDGVPGKG<br>VPG
**H_6-*B*^{GBP1}-*E*$^{S}_{3}$-*M*$^{HR00C\_3\_2}$-*E*$^{Z}_{80}$**	MGSSHHHHHHSSGMHGKTQATSGTIQSGSGVPGSGVPGSG<br>VPMIEEVVAEMIDILAESSKKSIEELARAADNKTTEKAVAE<br>AIEEIARLATAAIQLIEALAKNLASEEFMARAISAIAELAKK<br>AIEAIYRLADNHTTDTFMARAIAAIANLAVTAILAIAALASN

	HTTEEFMARAISAIAELAKKAIEAIYRLADNHTTDKFMAA<br>AIEAIALLATLAILAIALLASNHTTEEFMAKAISAIAELAKK<br>AIEAIYRLADNHTSPTYIEKAIEAIEKIARKAIKAIEMLAKNI<br>TTEEYKEKAKSAIDEIREKAKEAIKRLEDNRTGDGVPGKG<br>VPGDGVPGKGVPGDGVPGKGVPGDGVPGKGVPGDGVPG<br>KGVPGDGVPGKGVPGDGVPGKGVPGDGVPGKGVPGDGV<br>PGKGVPGDGVPGKGVPGDGVPGKGVPGDGVPGKGVPGD<br>GVPGKGVPGDGVPGKGVPGDGVPGKGVPGDGVPGKGVP<br>GDGVPGKGVPGDGVPGKGVPGDGVPGKGVPGDGVPGKG<br>VPGDGVPGKGVPGDGVPGKGVPGDGVPGKGVPGDGVPG<br>KGVPGDGVPGKGVPGDGVPGKGVPGDGVPGKGVPGDGV<br>PGKGVPGDGVPGKGVPGDGVPGKGVPGDGVPGKGVPGD<br>GVPGKGVPGDGVPGKGVPGDGVPGKGVPGDGVPGKGVP<br>GDGVPGKGVPGDGVPGKGVPGDGVPGKGVPGDGVPGKG<br>VPGDGVPGKGVPG

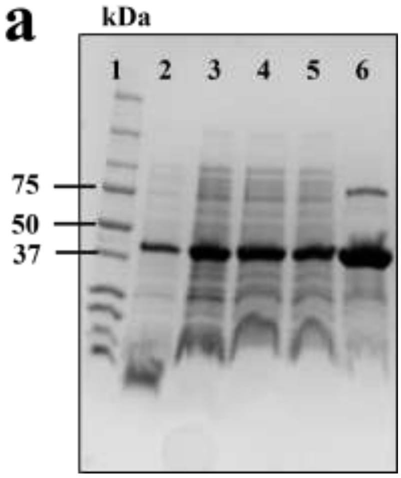

Figure S1. a) SDS-PAGE at various stages of purification of ***B-M-E***$_{20}$. From left to right, Lane 1: molecular weight marker; lane 2: intact cells; lane 3: cell lysate; lane 4: soluble lysate; lane 5: IMAC flow through; lane 6: IMAC fractions.

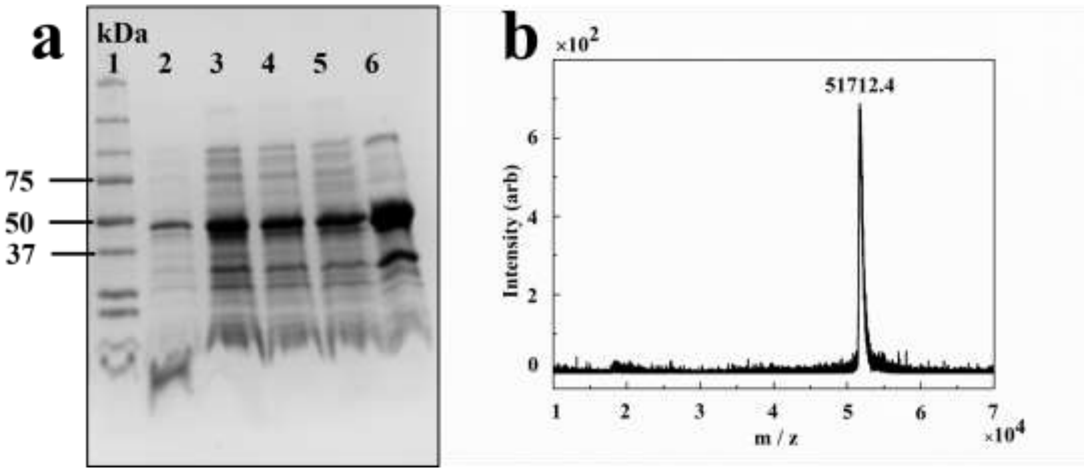

Figure S2. a) SDS-PAGE at various stages of purification of ***B-M-E***$_{40}$. From left to right, Lane 1: molecular weight marker; lane 2: intact cells; lane 3: cell lysate; lane 4: soluble lysate; lane 5: IMAC flow through; lane 6: IMAC fractions. b) MALDI-TOF mass spectrum for 1 mg/mL of ***B-M-E***$_{40}$.

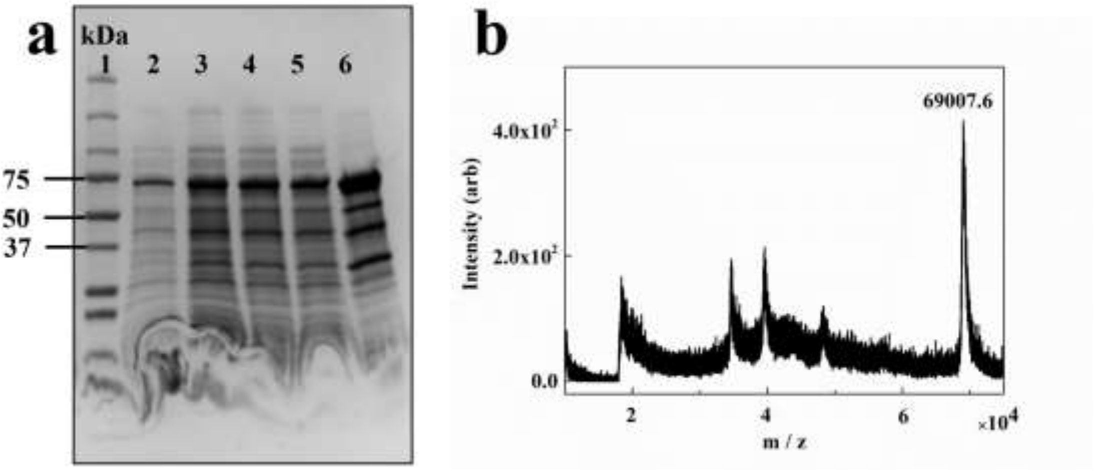

Figure S3. a) SDS-PAGE at various stages of purification of ***B*-*M*-*E***$_{80}$. From left to right, Lane 1: molecular weight marker; lane 2: intact cells; lane 3: cell lysate; lane 4: soluble lysate; lane 5: IMAC flow through; lane 6: IMAC fractions. b) MALDI-TOF mass spectrum for 1 mg/mL of ***B*-*M*-*E***$_{80}$.

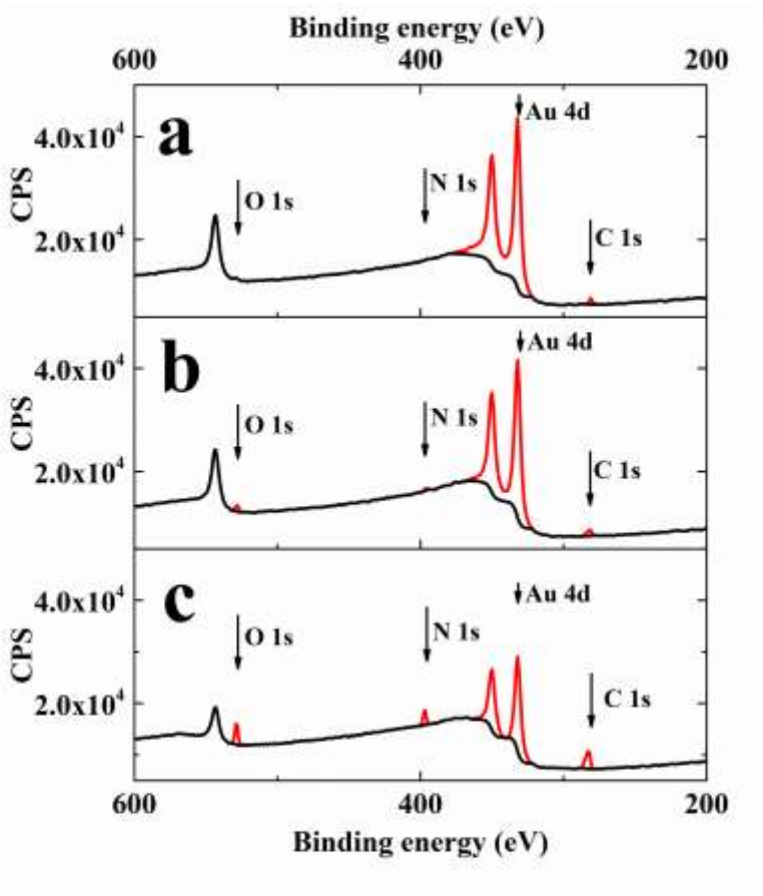

Figure S4. XPS results of different compounds on gold surfaces before ***B-M-E*** $_{20}$ coating and after coating. From top to bottom. a) Bare gold surface; b) 0.5 µM ***B-M-E*** $_{20}$ on gold surface for 3 s; c) 5 µM ***B-M-E*** $_{20}$ on gold surface for 5 min.

Table S4. Percentage of different compounds on gold surfaces before ***B-M-E*$_{20}$** coating and after coating.

	Percentage of **Au** 4d	Percentage of **C** 1s	Percentage of **O** 1s	Percentage of **N** 1s
Bare gold surface	85.9 %	14.1 %	0 %	0 %
0.5 µM ***B-M-E*$_{20}$** on gold surface for 3 s	65.6 %	23.6 %	5.2 %	5.6 %
5 µM ***B-M-E*$_{20}$** on gold surface for 5 min	27.6 %	44.8%	13.4 %	14.1 %

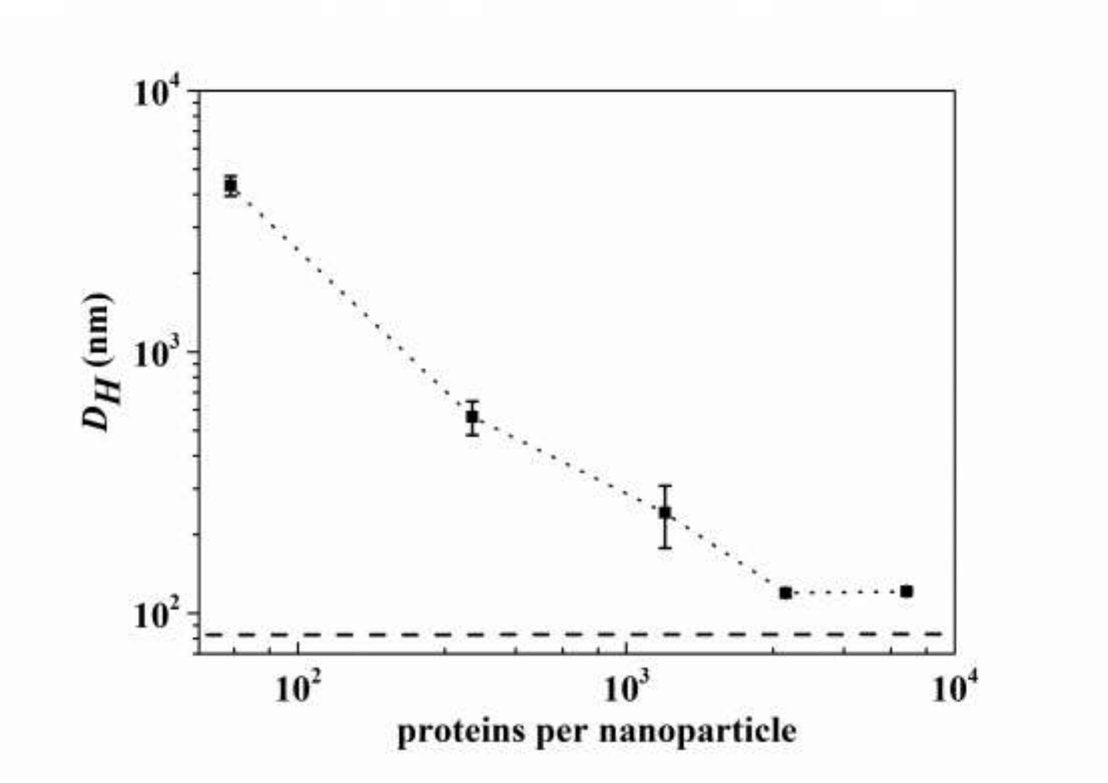

Figure S5. Interaction of ***B-M-E***$_{40}$ triblocks with d = 83 nm diameter gold particles. Effective hydrodynamic diameter (nm) of the particles as determined using Dynamic Light Scattering, versus proteins per nanoparticle in solution. Vertical dashed line represents the diameter (83 nm) of the bare gold particles. The diameter of ***B-M-E***$_{40}$ coated gold-nanoparticles is D = 119 nm, suggesting a layer thickness h for the ***B-M-E***$_{40}$ coating of $h = (D\text{-}d)/2$ = 18 nm. All DLS measurements are averages of 15 independent measurements.

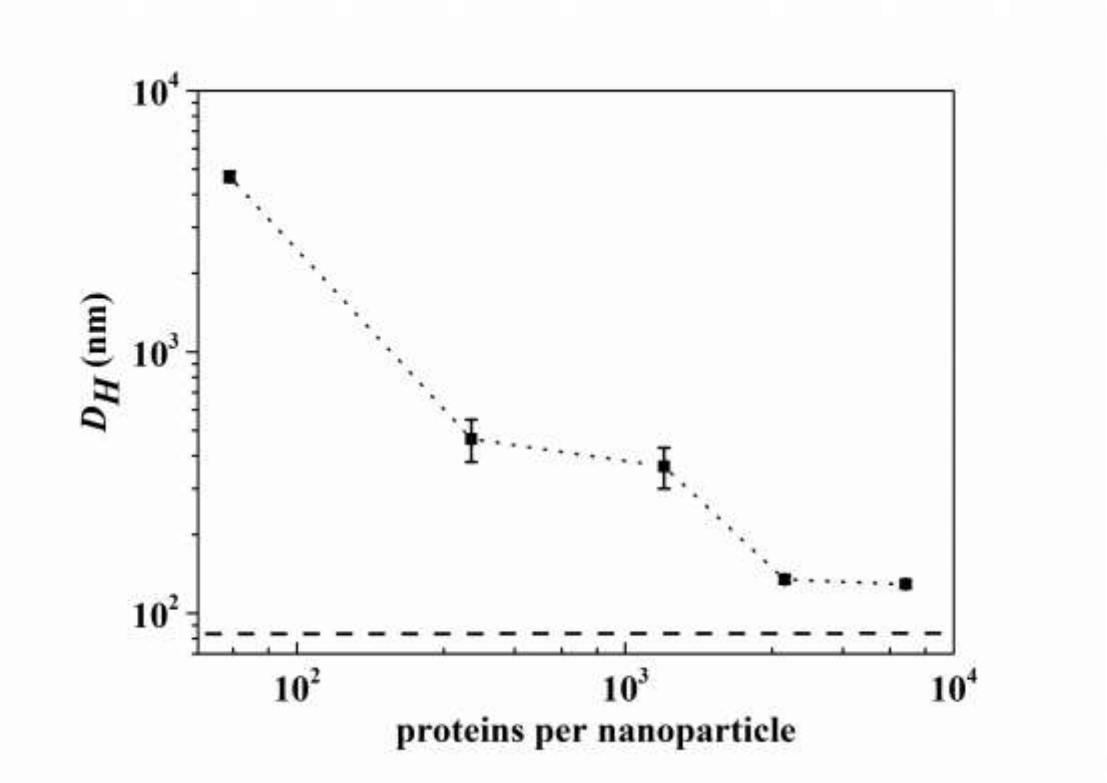

Figure S6. Interaction of ***B-M-E***$_{80}$ triblocks with d = 83 nm diameter gold particles. Effective hydrodynamic diameter (nm) of the particles as determined using Dynamic Light Scattering, versus proteins per nanoparticle. Dash line represents the diameter (83 nm) of the bare gold particles. The diameter of ***B-M-E***$_{80}$ coated gold-nanoparticles is D = 134 nm, suggesting a layer thickness h for the ***B-M-E***$_{80}$ coating of $h = (D-d)/2 = 25.5$ nm. All DLS measurements are averages of 15 independent measurements.

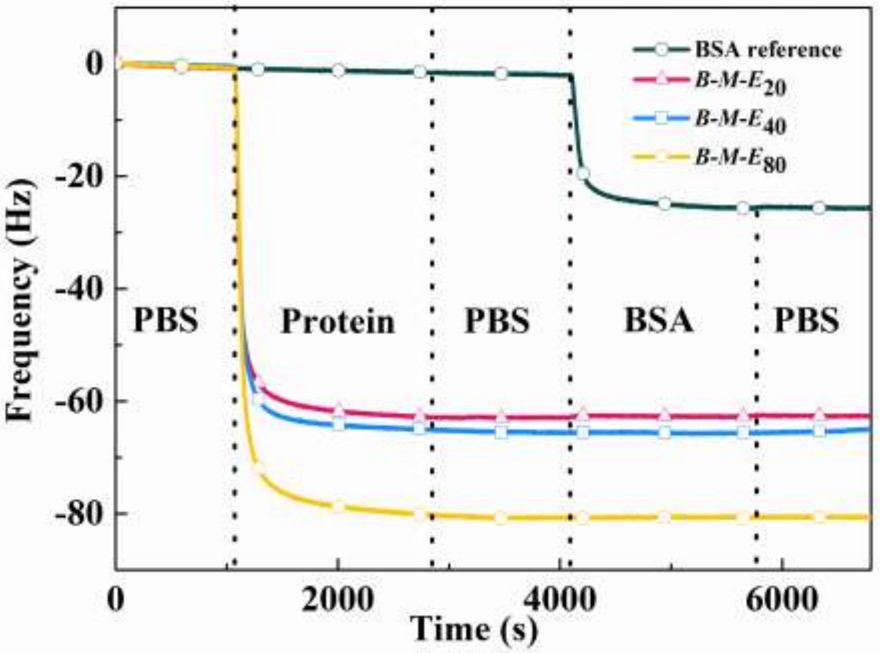

Figure S7. Additional QCM-D data of ***B-M-E*** antifouling properties, using Bovine Serum Albumin (BSA) as a general model protein foulant. QCM frequency shift (Hz) versus time t (s) after start of injection of 10 µM ***B-M-E*** protein. Red line with triangle, blue line with square and yellow line with pentagon are the ***B-M-E***$_{20}$, ***B-M-E***$_{40}$ and ***B-M-E***$_{80}$ binding to gold surface, respectively. Blue-green line with circle is the reference channel only injecting BSA with 1 mg/mL. At first, a flat base line was obtained by flushing with PBS; then protein with 10 µM were injected; after protein injection, PBS was injected again to rinse unbinded protein; then BSA solution was injected; at the end PBS was injected again.

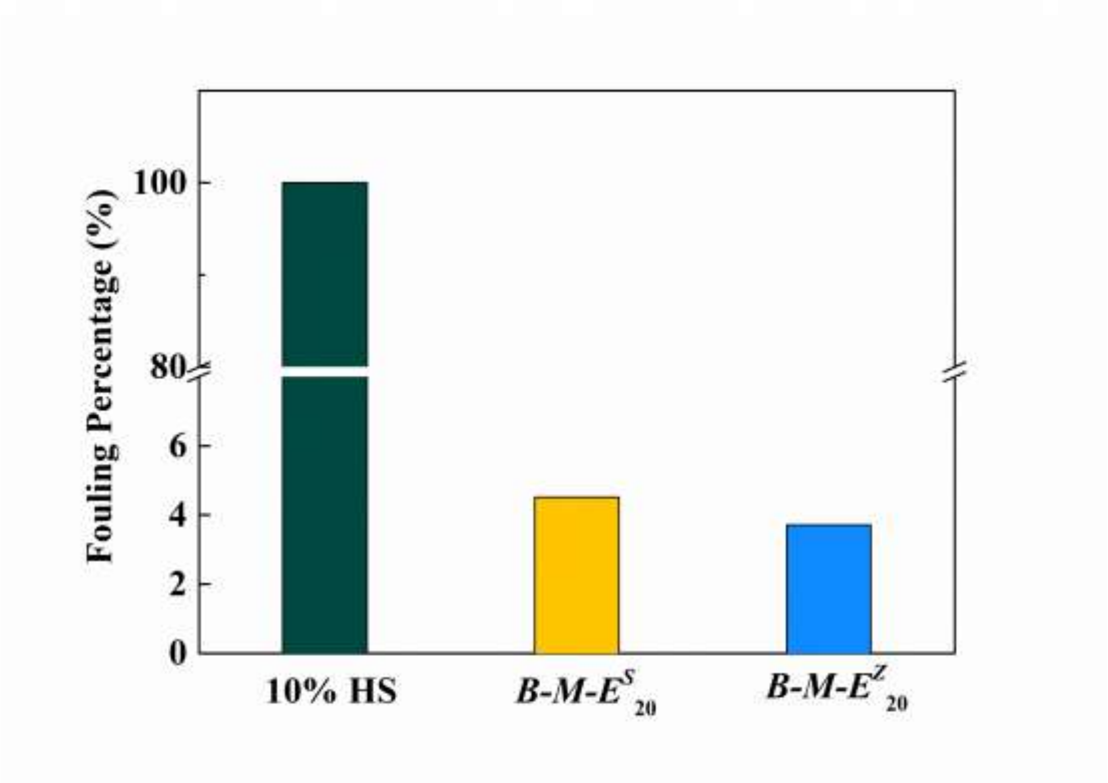

Figure S8. Effect of ***E*** block sequence on antifouling performance. Comparison between Serine (S) or alternating charged amino acids (K or D) as guest residue in the ELP blocks ***E***. Frequency change between the timepoints just before the injection of 10% HS and the end of the final rinse with PBS, normalized by the frequency change for the reference case (no coating, bare gold surface), in percentage, for $\boldsymbol{B\text{-}M\text{-}E^{S}}_{20}$ and $\boldsymbol{B\text{-}M\text{-}E^{Z}}_{20}$.

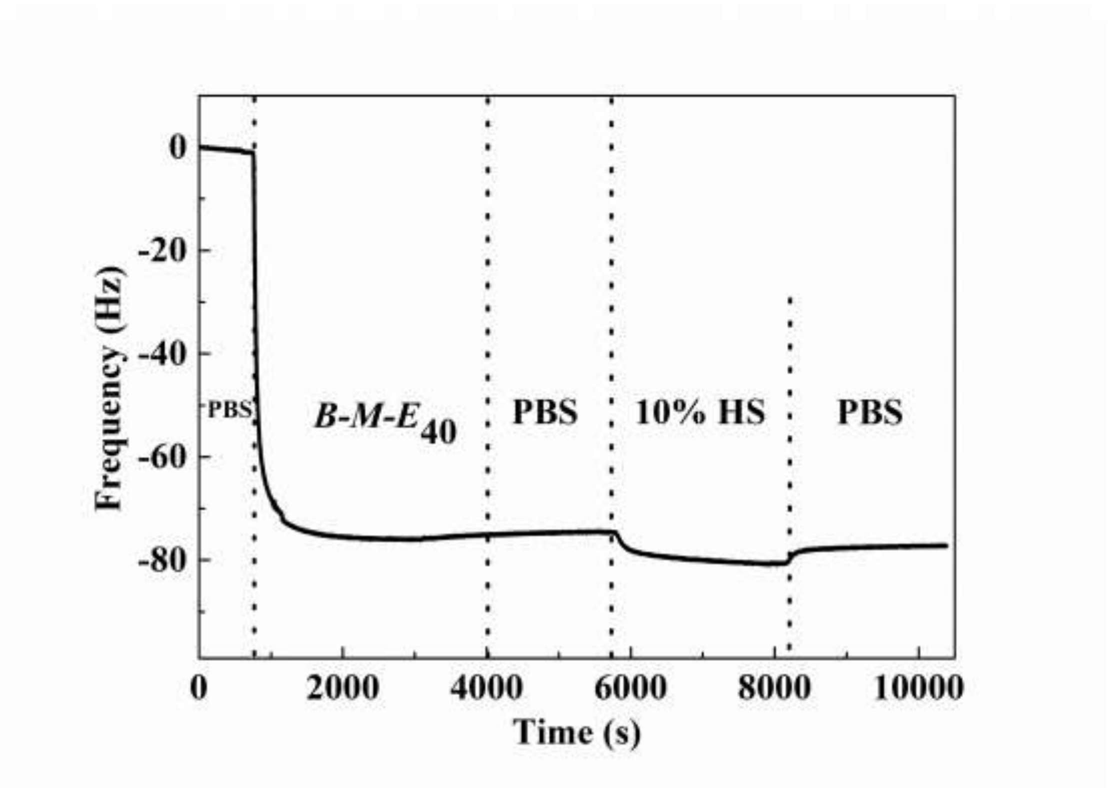

Figure S9. QCM-D assay of antifouling performance of ***B-M-E***$_{40}$ against 10% HS for longer incubation times. QCM frequency shift (Hz) versus time t (s) after start of injection of 10 μM ***B-M-E***$_{40}$ protein.

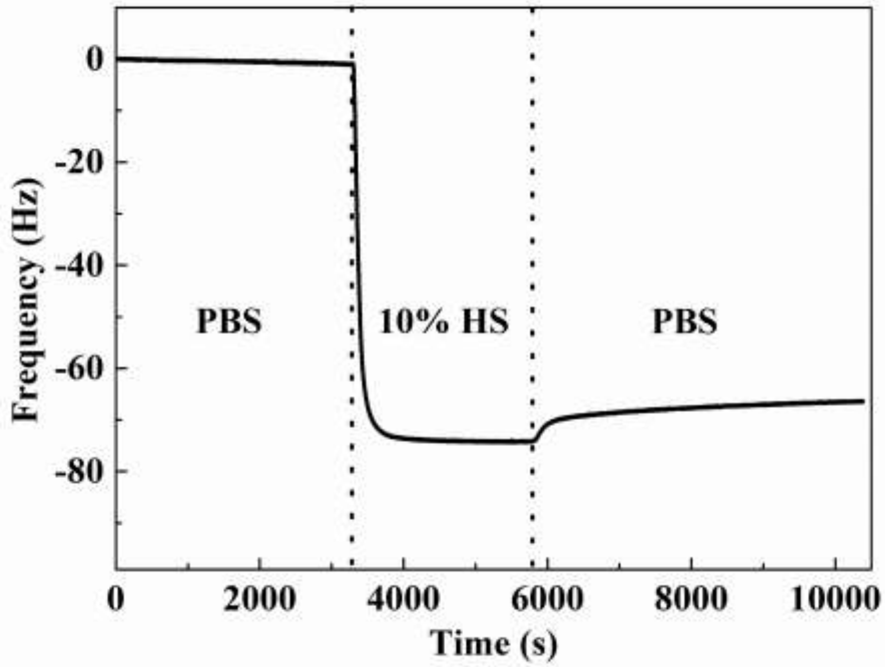

Figure S10. QCM-D assay of 10%HS – reference case of no ***B-M-E*** coating. QCM frequency shift (Hz) versus time t (s) after start of injection of 10% HS.

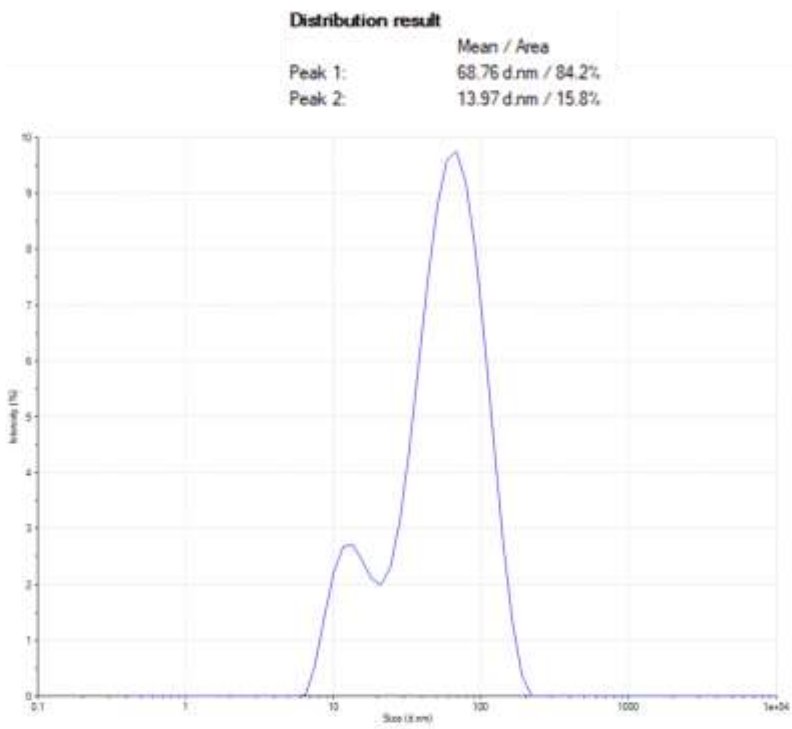

Figure S11. Interaction of ***B-M-E*$_{20}$** triblocks with smaller d = 42 nm diameter gold nano particles (GNP). Distribution plot of effective hydrodynamic diameter (nm) versus scattering intensity as obtained from Dynamic Light Scattering. Excess protein was added to GNPs. The largest peak at D = 69 nm corresponds to the fully coated GNP and suggests a layer thickness $h = (D\text{-}d)/2$ = 13.5 nm. The smaller peak at D = 14 nm corresponds to the solution size of the dissolved ***B-M-E*$_{20}$** trimers. All DLS measurements are averages of 15 independent measurements.

(1) Alvisi, N.; Zheng, C.; Lokker, M.; Boekestein, V.; de Haas, R.; Albada, B.; de Vries, R. Design of Polypeptides Self-Assembling into Antifouling Coatings: Exploiting

Multivalency. *Biomacromolecules* **2022**, *23* (9), 3507–3516. https://doi.org/10.1021/acs.biomac.2c00170.
